# Supplementary material for: Reward-Based Learning Drives Rapid Sensory Signals in Medial Prefrontal Cortex and Dorsal Hippocampus Necessary for Goal-Directed Behavior
Source: Neuron. 2018 Jan 3;97(1):83–91.e5. doi: 10.1016/j.neuron.2017.11.031 (PMC5766832; doi:10.1016/j.neuron.2017.11.031)
Supplement: Document S1. Figures S1–S4 [file mmc1.pdf]

Neuron, Volume *97*

Supplemental Information

Reward-Based Learning Drives Rapid Sensory Signals in Medial Prefrontal Cortex and Dorsal Hippocampus Necessary for Goal-Directed Behavior

Pierre Le Merre, Vahid Esmaeili, Eloïse Charrière, Katia Galan, Paul-A. Salin, Carl C.H. Petersen, and Sylvain Crochet

Reward-based learning drives rapid sensory signals in medial prefrontal cortex and dorsal hippocampus necessary for goal-directed behavior

Pierre Le Merre, Vahid Esmaeili, Eloïse Charière, Katia Galan, Paul-A. Salin, Carl C.H. Petersen and Sylvain Crochet

Supplemental Information consists of:

Figure S1, related to Figure 1
Figure S2, related to Figure 1
Figure S3, related to Figure 1
Figure S4, related to Figure 4

Figure S1

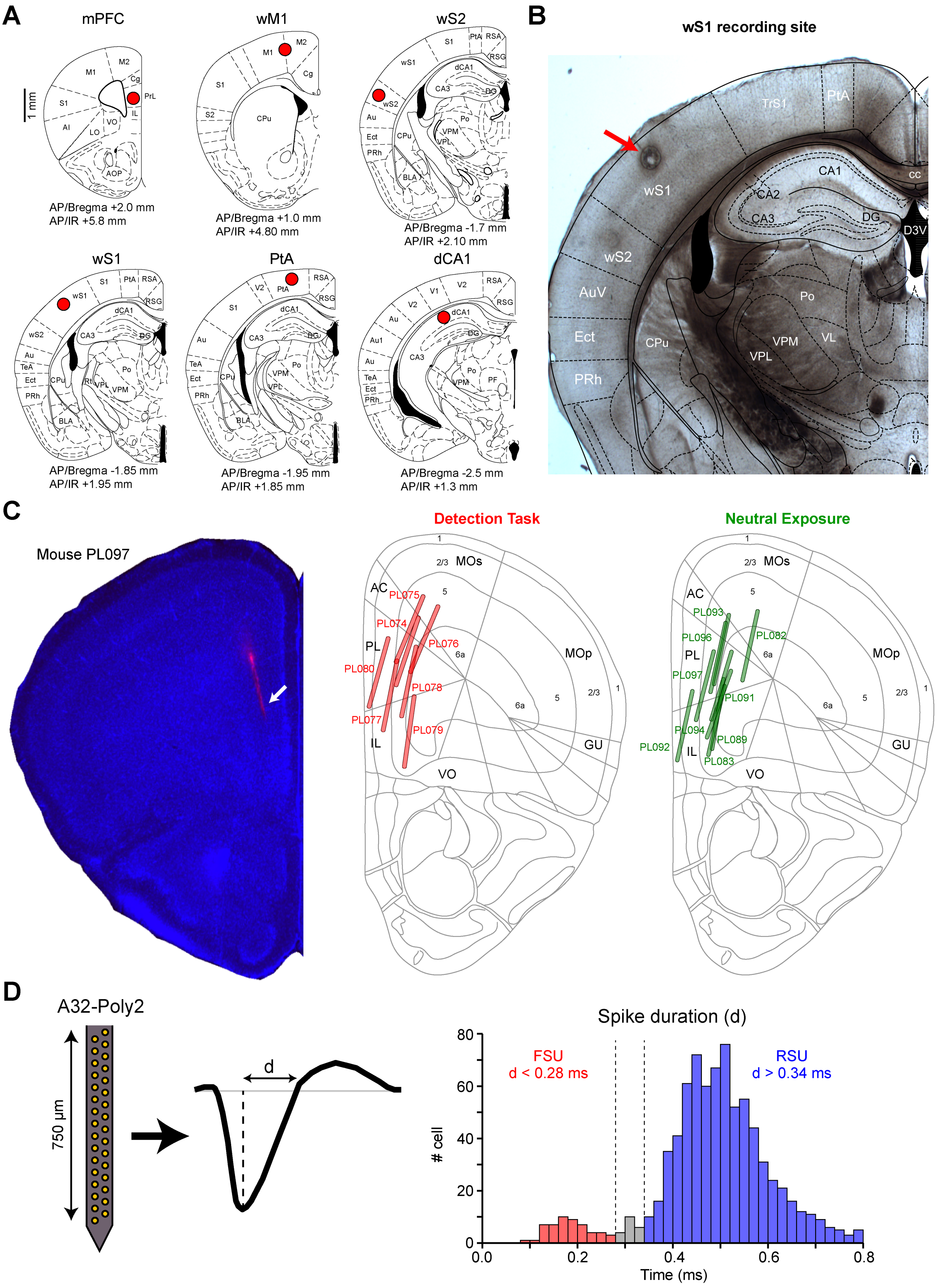

Figure S1. LFP and high-density extracellular recordings

(A) Schematic representation of the targeted cortical areas for multisite LFP recordings. From anterior to posterior: mPFC, wM1, wS2, wS1, PtA and dCA1. Anterio-posterior

coordinates relative to Bregma (AP/Bregma) and interaural (AP/IR) are indicated below each site.

(B) Example anatomical verification of a wS1 LFP recording site. At the end of the experiment, electrolytic lesions were performed under anesthesia for each LFP electrode, before perfusion. 100 µm thick sections were cut to identify recording sites.

(C) *Left*, Example anatomical verification of a silicon-probe recording site in mPFC. The probe was coated with DiI. At the end of the experiment the mouse was perfused. 100 µm thick sections were cut to identify recording sites. *Right*, Location of the silicon-probes in the mPFC of Trained (red) and Exposed (green) mice.

(D) *Left*, Schematic representation of the A32-Poly2 Neuronexus probe used for mPFC extracellular recordings and spike waveform. The duration of the extracellular action-potential (d) was computed as the time between the peak and return-to-baseline. *Right*, Distribution of spike duration for all mPFC recorded units. Units with spike duration below 0.28 ms were classified as fast-spiking units (FSUs, red). Units with spike duration above 0.34 ms were classified as regular-spiking units (RSUs, blue).

Figure S2

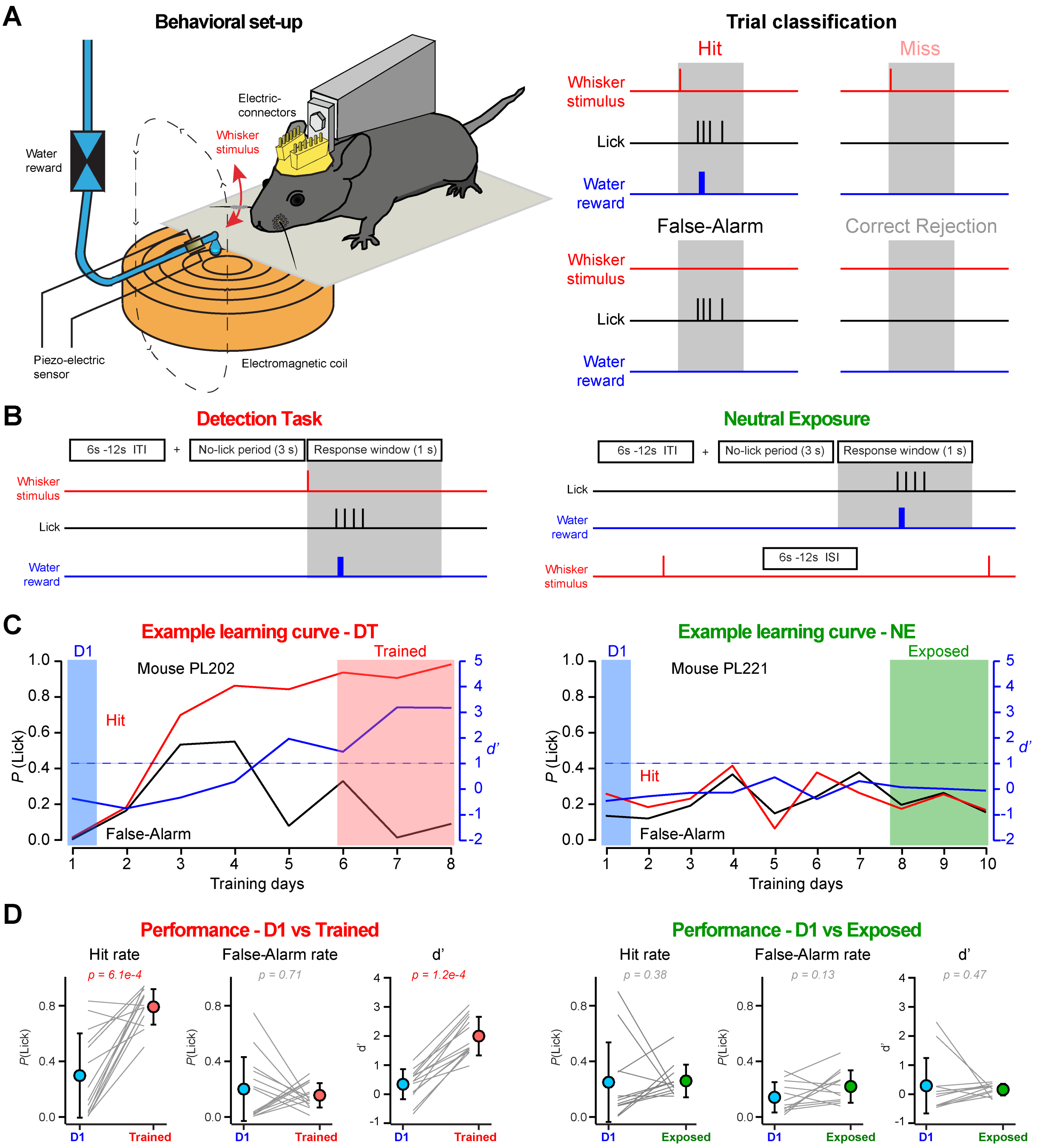

Figure S2. Detection task and neutral exposure

(A) *Left*, Schematic drawing of the behavioral set-up. *Right*, Trial classification. For Neutral Exposure, Licking within the 1 s window following whisker stimulus was scored as Hit, regardless of reward delivery.

(B) Schematic representation of the trial time-line of the two behavioral tasks: detection task (left) and neutral exposure (right). In the detection task, whisker stimulation predicted reward availability. In neutral exposure training, whisker stimulation did not predict reward availability and whisker stimuli were delivered asynchronously at random times.

(C) Example learning curves for a mouse trained in the detection task (*Left*) and a mouse during neutral exposure (*Right*). The red and black curves indicate lick probability (P Lick) on Stimulus trials (Hit rate) and Catch trials (False-Alarm rate), respectively. The blue curve indicates d'.

(D) Performance of all the mice used for LFP recordings in the detection task (*Left*) and neutral exposure (*Right*) for the first session (D1) and mean of the three trained or exposed sessions. Values are mean ± SD. Red *p* values indicate statistically significant differences between D1 and Trained or Exposed (Wilcoxon Signed-Rank test).

Figure S3

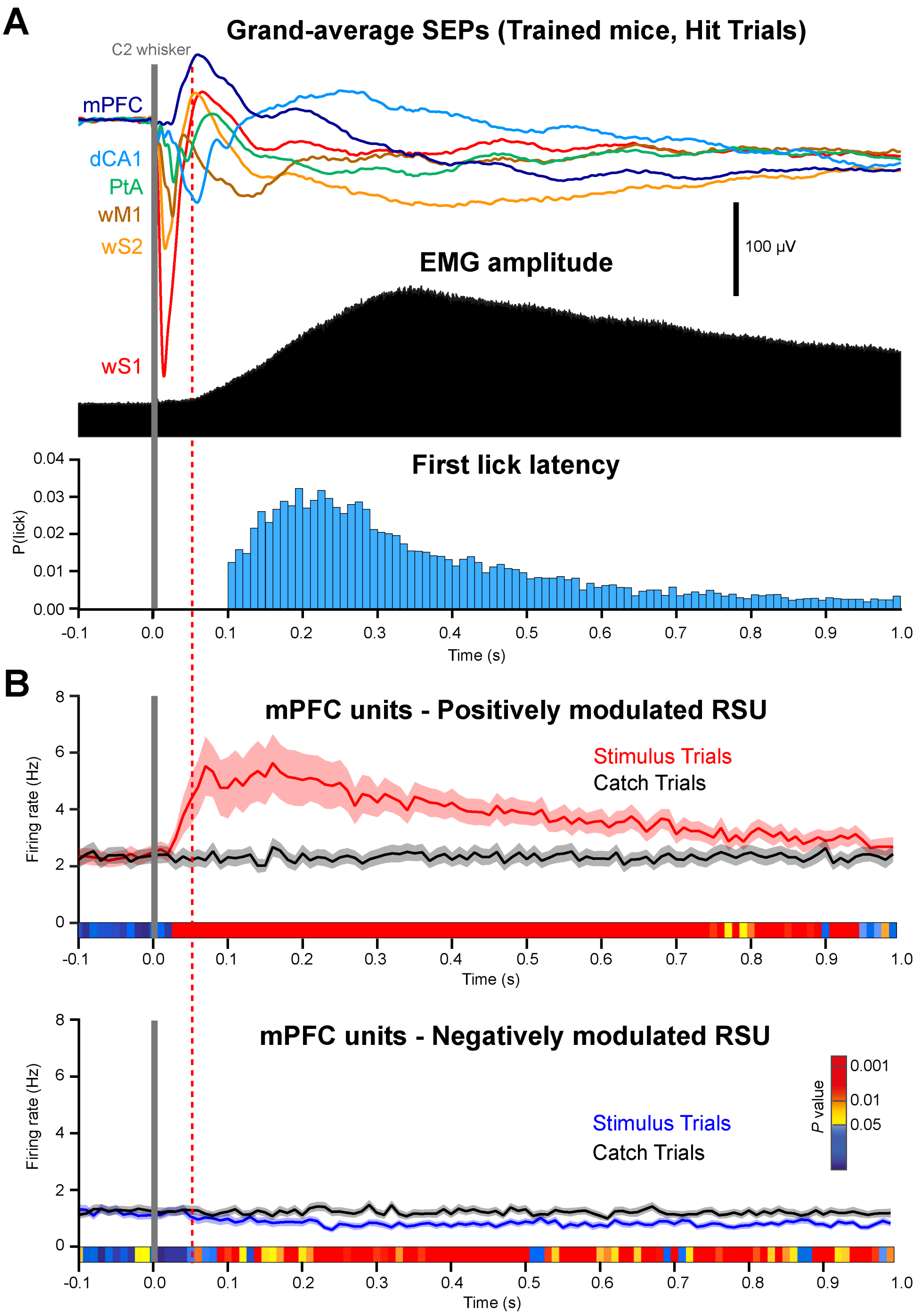

Figure S3. Sensory evoked responses precede EMG activity and licking

(A) *Top*, Grand-average SEPs from the 6 cortical areas computed for Hit trials in trained mice. Premature (<100 ms) lick trials have been excluded. *Middle*, Grand-average EMG amplitude. *Bottom*, Distribution of the first-lick latency. The red dashed line indicates the beginning of the EMG activation.

(B) *Top*, Average PSTHs from mPFC computed for positively (*above*) and negatively (*below*) modulated units for all Stimulus trials (Red and Blue) and all Catch trials (Black). Shaded areas indicate SEM. The bar below the PSTHs indicates color-coded *p* values for statistical test of the difference between Stimulus and Catch trials across time (Wilcoxon signed-rank test Stimulus vs Catch for 10 ms time windows).

Figure S4

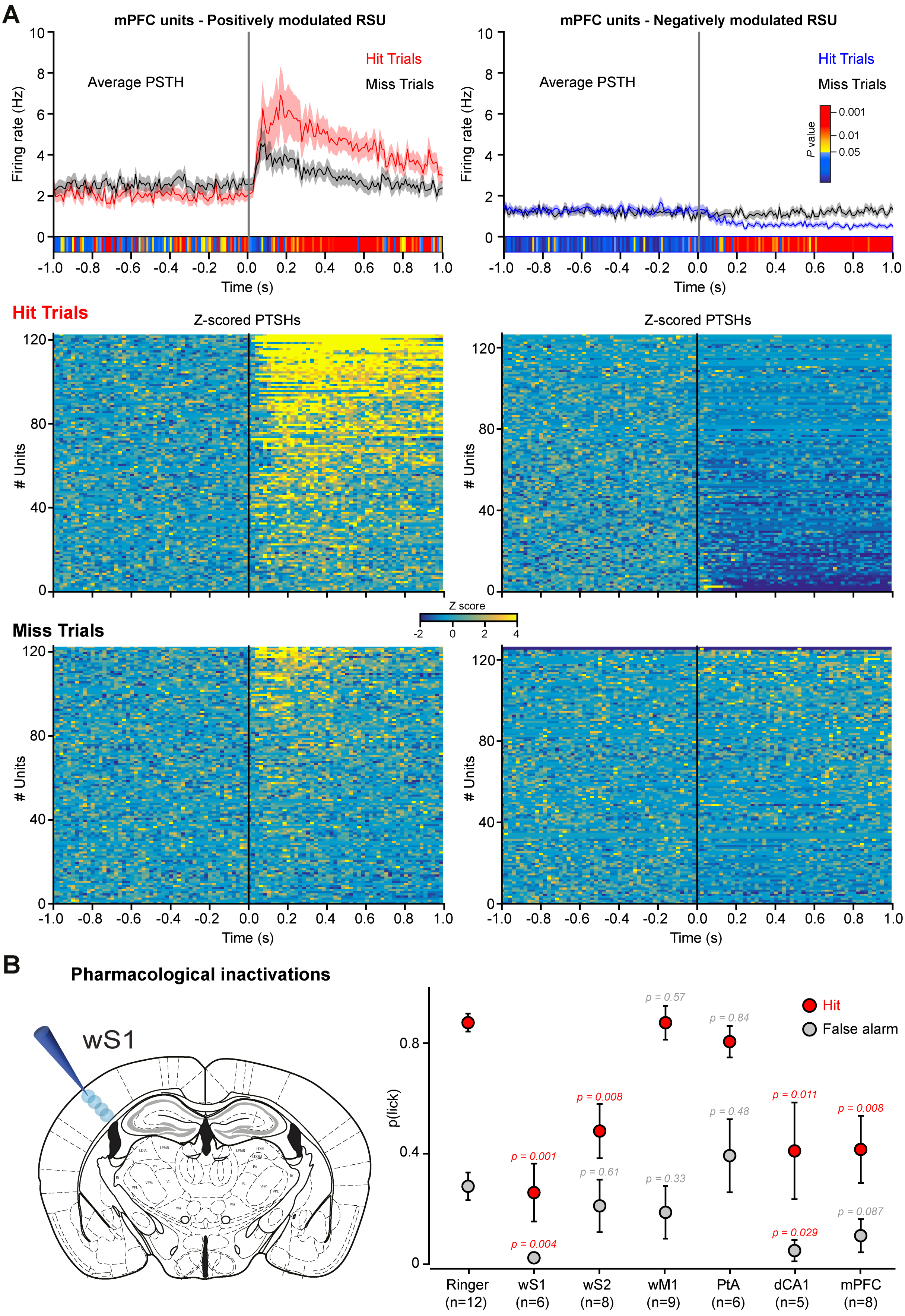

A
mPFC units - Positively modulated RSU
mPFC units - Negatively modulated RSU
Average PSTH
Hit Trials
Miss Trials
Firing rate (Hz)
P value
0.001
0.01
0.05
Time (s)
Hit Trials
Z-scored PTSHs
Units
Z score
Miss Trials
B
Pharmacological inactivations
wS1
p(lick)
Hit
False alarm
p = 0.57
p = 0.84
p = 0.008
p = 0.011
p = 0.008
p = 0.001
p = 0.61
p = 0.48
p = 0.33
p = 0.004
p = 0.029
p = 0.087
Ringer (n=12)
wS1 (n=6)
wS2 (n=8)
wM1 (n=9)
PtA (n=6)
dCA1 (n=5)
mPFC (n=8)

Figure S4. Neuronal activity in mPFC and dCA1 causally contributes to detection task execution

(A) *Top*, Average PSTHs computed for positively (*Left*) and negatively (*Right*) modulated units for Hit (Red and Blue) and Miss (Black) trials. Shaded areas indicate SEM. The bar below the PSTHs indicates *p* values color-coded as indicated in the inset for statistical test of the difference between Hit and Miss trials across time (Wilcoxon Signed-Rank test Hit vs Miss for 10 ms time windows). *Below*, heat-maps of the z-scored PSTHs. The z-scored PSTHs of individual units (20 ms bin size) were aligned to stimulus onset and sorted according to their change in firing rate relative to baseline.

(B) *Left*, Schematic drawing representing the pharmacological inactivation protocol in wS1. *Right*, Effect of pharmacological inactivation (Muscimol, 5 mM, 4 x or 5 x 100 nl) on detection task performance (Hit rate, red; False-Alarm, grey) for each area and comparison with control Ringer injections. The number of mice for each area is indicated in parenthesis. Values are mean ± SD. Statistical significance was assessed by comparing the effect of Muscimol injection with control Ringer injections using Mann-Whitney two sample rank test with a Bonferroni-Holm correction for multiple comparisons: red *p* values indicate statistically significant differences.
