# Supplementary material for: Reward-Based Learning Drives Rapid Sensory Signals in Medial Prefrontal Cortex and Dorsal Hippocampus Necessary for Goal-Directed Behavior
Source: Neuron. 2018 Jan 3;97(1):83–91.e5. doi: 10.1016/j.neuron.2017.11.031 (PMC5766832; doi:10.1016/j.neuron.2017.11.031)
Supplement: Document S2. Article plus Supplemental Information [file mmc2.pdf]

Neuron

Report

Reward-Based Learning Drives Rapid Sensory Signals in Medial Prefrontal Cortex and Dorsal Hippocampus Necessary for Goal-Directed Behavior

Highlights

- Sensory-evoked responses in mPFC and dCA1 develop during learning
- Sensory processing in mPFC and dCA1 is fast, beginning within 50 ms of stimulus
- Sensory responses in mPFC and dCA1 correlate trial by trial with performance
- mPFC and dCA1 are necessary for execution of a sensory detection task

Authors

Pierre Le Merre, Vahid Esmaeili, Eloïse Charrière, Katia Galan, Paul-A. Salin, Carl C.H. Petersen, Sylvain Crochet

Correspondence

carl.petersen@epfl.ch (C.C.H.P.), sylvain.crochet@epfl.ch (S.C.)

In Brief

Le Merre et al. investigate a whisker-dependent detection task, revealing that learning is accompanied by development of fast sensory processing in medial prefrontal cortex and dorsal hippocampus, and that neuronal activity in these brain regions is required for task execution.

Le Merre et al., 2018, Neuron *97*, 83–91
January 3, 2018 © 2017 The Author(s). Published by Elsevier Inc.
https://doi.org/10.1016/j.neuron.2017.11.031

CellPress

Neuron
Report

CellPress

Reward-Based Learning Drives Rapid Sensory Signals in Medial Prefrontal Cortex and Dorsal Hippocampus Necessary for Goal-Directed Behavior

Pierre Le Merre,[1,2,3] Vahid Esmaeili,[1] Eloïse Charrière,[1] Katia Galan,[1] Paul-A. Salin,[2] Carl C.H. Petersen,[1,*] and Sylvain Crochet[1,3,4,*]
[1]Laboratory of Sensory Processing, Brain Mind Institute, Faculty of Life Sciences, Ecole Polytechnique Fédérale de Lausanne (EPFL), Lausanne CH-1015, Switzerland
[2]Lyon Neuroscience Research Center, INSERM U1028; CNRS UMR5292; University Lyon 1, Forgetting and Cortical Dynamics Team, Lyon Cedex 08 F-69000, France
[3]Lyon Neuroscience Research Center, INSERM U1028; CNRS UMR5292; University Lyon 1, Integrative Physiology of the Brain Arousal System Team, Lyon Cedex 08 F-69000, France
[4]Lead Contact
*Correspondence: carl.petersen@epfl.ch (C.C.H.P.), sylvain.crochet@epfl.ch (S.C.)
https://doi.org/10.1016/j.neuron.2017.11.031

SUMMARY

The neural circuits underlying learning and execution of goal-directed behaviors remain to be determined. Here, through electrophysiological recordings, we investigated fast sensory processing across multiple cortical areas as mice learned to lick a reward spout in response to a brief deflection of a single whisker. Sensory-evoked signals were absent from medial prefrontal cortex and dorsal hippocampus in naive mice, but developed with task learning and correlated with behavioral performance in mice trained in the detection task. The sensory responses in medial prefrontal cortex and dorsal hippocampus occurred with short latencies of less than 50 ms after whisker deflection. Pharmacological and optogenetic inactivation of medial prefrontal cortex or dorsal hippocampus impaired behavioral performance. Neuronal activity in medial prefrontal cortex and dorsal hippocampus thus appears to contribute directly to task performance, perhaps providing top-down control of learned, context-dependent transformation of sensory input into goal-directed motor output.

INTRODUCTION

The neural circuits involved in transforming relevant sensory information into goal-directed motor output are poorly understood. In the absence of engagement in a behavioral task or even under anesthesia, sensory stimuli drive neural activity in multiple brain areas through innate feedforward signaling pathways. This first level of cortical sensory processing occurs predominantly in primary and secondary sensory areas, but also in related motor areas. For example, in the anesthetized mouse, a single-whisker deflection evokes a sensory response that initiates in the primary whisker somatosensory cortex (wS1) and rapidly spreads to the secondary whisker somatosensory cortex (wS2) and the primary whisker motor cortex (wM1) (Ferezou et al., 2007; Mohajerani et al., 2013).

Sensory signals can become behaviorally important through learning, in which case they need to be routed to the appropriate motor circuits in order to contribute to goal-directed behavior. The transformation of external sensory signals into learned motor output appears to occur across a large network of brain areas that include sensory and motor neocortex, as well as associative and higher-order areas (Romo and de Lafuente 2013; Guo et al., 2014; Siegel et al., 2015). Among the associative and higher-order areas, the associative parietal area (PtA), the medial prefrontal cortex (mPFC), and the dorsal CA1 region of the hippocampus (dCA1) have been found to be implicated in different goal-directed behaviors in rodents, including contextual and spatial memory (Benchenane et al., 2010; Harvey et al., 2012; Place et al., 2016), multisensory integration (Song et al., 2017; Aronov et al., 2017; Terada et al., 2017), and sensory detection (Pinto and Dan, 2015; Otis et al., 2017) or discrimination (Pereira et al., 2007; Itskov et al., 2011).

Yet the respective roles of sensory and higher-order cortical areas in sensory processing and goal-directed behavior remain poorly understood. Here, to address this issue, we made electrophysiological recordings from different sensory and higher-order cortical areas while mice were trained in a detection task in which whisker deflection predicted reward availability (Sachidhanandam et al., 2013; Sippy et al., 2015; Yamashita and Petersen, 2016), or were exposed to the same whisker stimulus that was not rewarded. We also used local optogenetic and pharmacological inactivation to assess the necessity of the recorded areas for the execution of the detection task.

RESULTS

We longitudinally monitored neural activity simultaneously from six cortical areas using chronic multisite local field potential (LFP) recordings (Figures 1A and S1; STAR Methods) (Fernandez et al., 2017) while mice learned a whisker-based sensory

This is an open access article under the CC BY license (http://creativecommons.org/licenses/by/4.0/).

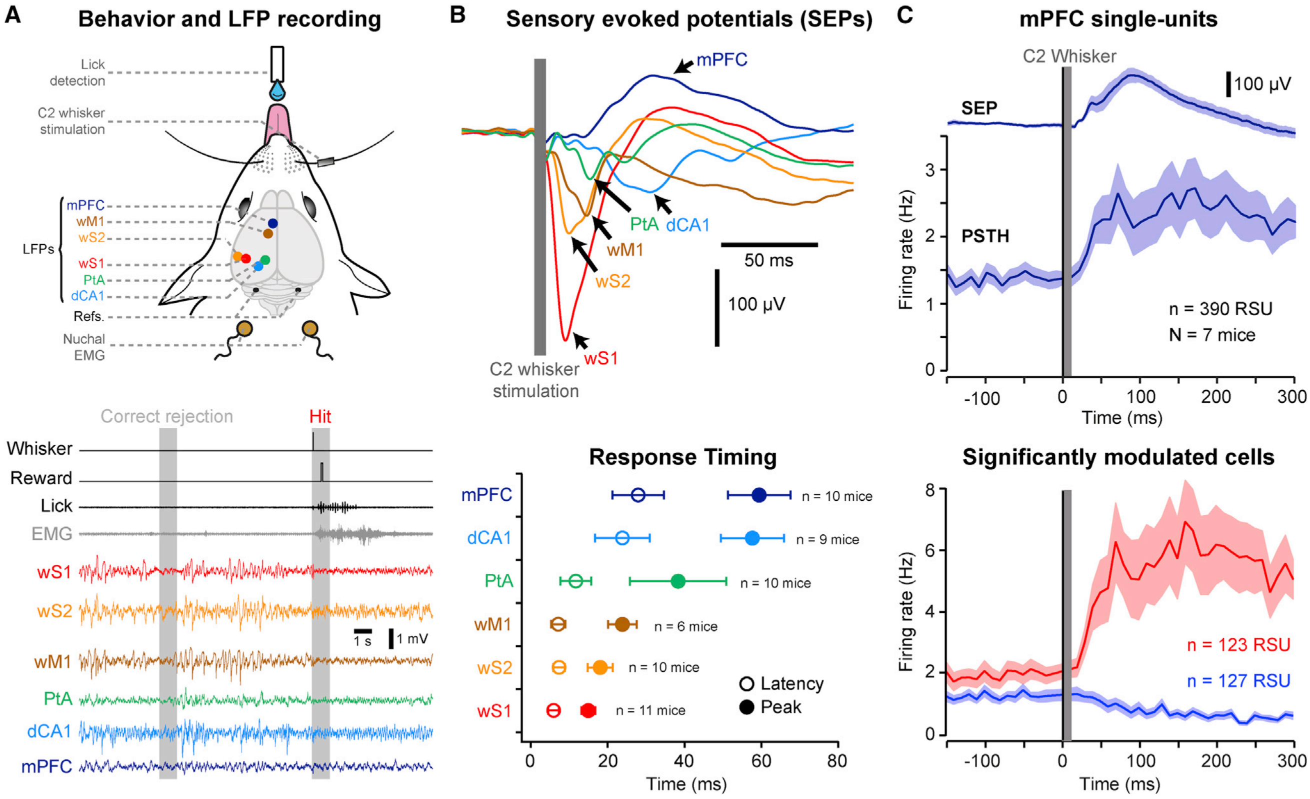

Figure 1. Sensory-Evoked Responses during the Detection Task

(A) Top: schematic drawing showing the relative position of LFP electrodes in the dorsal CA1 subfield of the hippocampus (dCA1), the associative parietal area (PtA), the whisker fields of the primary and secondary somatosensory cortex (wS1 and wS2), the whisker field of the primary motor cortex (wM1), and the medial prefrontal cortex (mPFC). Nuchal EMG, nuchal electromyogram; Refs, reference and ground wires in the cerebellum. Bottom: example simultaneous recording of LFPs (band-pass filtered 0.5–100 Hz), EMG, and behavioral signals during the detection task, including a Catch trial (Correct rejection) and a Stimulus trial (Hit). Whisker, pulse sent to the electromagnetic coil for whisker stimulation; Reward, pulse sent to open the valve that delivers water reward; Lick, signal generated by piezo film attached to the water spout to monitor licking.

(B) Top: grand average sensory-evoked potentials (SEPs) from the six cortical areas computed for Hit trials in trained mice. Premature (<100 ms) lick trials have been excluded. Bottom: mean latency (open circles) and peak time (filled circles) of the sensory-evoked potentials in the different cortical areas. Values are mean ± SD.

(C) Silicon probe recording in mPFC. Top: grand average SEP and PSTH computed for Hit trials from 390 regular spiking units (RSUs) recorded in mPFC of seven mice performing the detection task. Shaded areas indicate SEM. Bottom: PSTHs computed separately for the 123 positively (red) and 127 negatively (blue) significantly modulated units.

detection task during daily training sessions (Figure S2). In this task, water-restricted mice were rewarded with a drop of water if they licked a spout within the 1 s reward window that immediately followed a brief single-whisker deflection. Trials occurred at random times at 6–12 s intertrial intervals without preceding cues. Catch trials, in which no whisker stimulus was applied, were randomly interleaved to assess the False-Alarm rate (Figure S2). Mice learned the detection task in 5–12 days, reaching good performance (Hit rate = 0.79 ± 0.13, False-Alarm rate = 0.16 ± 0.09 and d' = 2.00 ± 0.68; mean ± SD, n = 14 mice) (Figure S2).

We first investigated which cortical areas were recruited during the execution of the detection task after learning. We averaged the sensory-evoked responses for each area (Figure 1B) for hit trials in trained mice. We found a fast and sequential recruitment of all recorded areas within the first 50 ms following whisker stimulus that preceded the behavioral response (Figure S3). Sensory areas (wS1, wS2, and wM1) were recruited significantly before dCA1 and mPFC (Dunn-Holland Wolfe test, $p < 0.05$). While the first sensory-evoked response had a negative polarity in most cortical areas compatible with a depolarizing response of the membrane potential, the sensory-evoked response in mPFC had a positive polarity. To clarify the nature of the mPFC response, we performed acute high-density extracellular action potential recordings using silicon probes (Buzsáki, 2004; Rossant et al., 2016) in another group of seven mice performing the detection task (Figure S1). After spike sorting, we isolated 390 regular spiking units (RSUs) located in both prelimbic and infralimbic areas of the mPFC (Figure S1). The grand average peristimulus time histogram (PSTH) showed a net increase in the activity of RSUs with short latency (<50 ms) after the whisker stimulus, indicating an overall excitatory response (Figures 1C and S3B). However, at the single-cell level we observed both positively and negatively

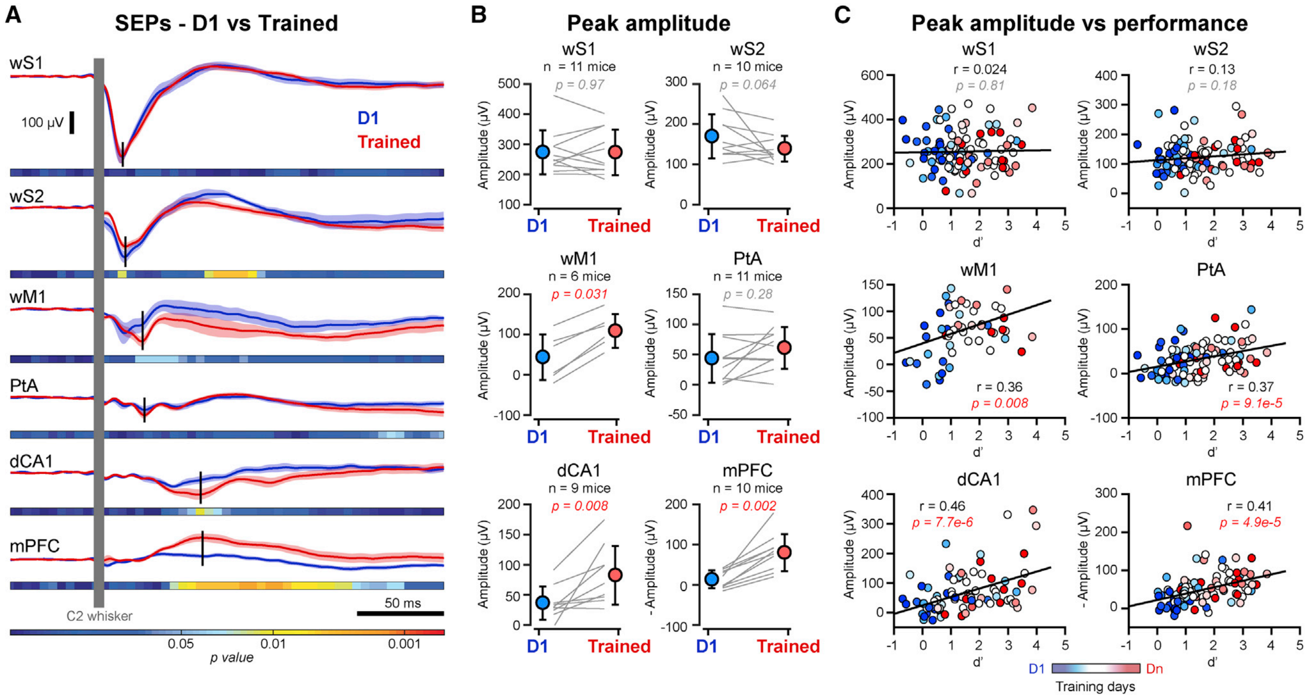

Figure 2. Sensory-Evoked Responses Correlate with Learning

(A) Grand average SEPs computed for all stimulus trials, on the first training day (D1, blue) and after reaching good performance (Trained, red) for the same mice. Shaded areas indicate SEM. Horizontal bars indicate color-coded p values for statistical test of the difference between D1 and Trained conditions across time (Wilcoxon signed-rank test, D1 versus Trained for 5 ms time windows). Vertical black bars indicate the times of the peak responses.

(B) Comparison of the peak amplitude of SEPs for D1 (blue) and Trained (red) conditions. Circles with bars indicate mean ± SD. Gray lines represent individual mice. Red p values indicate statistically significant differences between D1 and Trained (Wilcoxon signed-rank test).

(C) Scatterplots of the day-by-day mean peak amplitude of the SEPs against performance (d') for the six recorded areas. Each point represents one animal and one training session, color-coded according to the training day (from blue for D1 to red for the last training day). The correlation between the amplitude of the SEP and the daily performance was assessed using Pearson correlation with *t* statistic: the coefficient of correlation (*r*) and p values are indicated for each scatterplot.

modulated units, with 31.5% of the cells significantly increasing and 32.6% significantly decreasing firing rate following the whisker stimulus (Figure 1C).

In order to examine the impact of learning, we next compared the sensory-evoked activity in trained mice to the responses recorded in the same cortical areas from the same mice on the first day of training (D1). Interestingly, we found that the sensory-evoked response in mPFC and dCA1 were initially very small in amplitude or even absent, and developed during learning, showing a significant increase at the end of training (Trained) (Wilcoxon signed-rank test, D1 versus Trained: mPFC, $p = 0.002$; dCA1, $p = 0.008$) (Figures 2A and 2B). In contrast, the whisker-deflection-evoked responses in sensory areas (wS1 and wS2) remained rather stable throughout the training (Wilcoxon signed-rank test, D1 versus Trained: wS1, $p = 0.97$; wS2, $p = 0.064$) (Figures 2A and 2B). In line with this observation, the peak amplitude of the sensory-evoked response was best correlated with task performance (d') across training days in mPFC and dCA1 (Pearson correlation with t statistic: mPFC, $r = 0.41$, $p = 4.9 \times 10^{-5}$; dCA1, $r = 0.46$, $p = 7.7 \times 10^{-6}$), although significant correlations were also observed for wM1 and PtA (wM1, $r = 0.36$, $p = 0.008$; PtA, $r = 0.37$, $p = 9.1 \times 10^{-5}$), but not for wS1 and wS2 (wS1, $r = 0.024$, $p = 0.81$; wS2, $r = 0.13$, $p = 0.18$) (Figure 2C). Thus, the early recruitment of mPFC and dCA1 in response to whisker stimulus developed during training and correlated with learning. Yet this could simply result from the repetitive exposure to the same sensory stimulus over days. To address the specificity of the development of the evoked response in mPFC and dCA1, we longitudinally recorded the neural activity in the same six cortical areas in another group of mice ($n = 12$ mice) that were exposed to the same whisker stimulus, in the same context but without any temporal correlation between the whisker stimulus and reward delivery (Neutral Exposure; Figure S2). In this condition, the mice did not learn any association between whisker stimulus and reward delivery (Figure S2). Comparing the sensory-evoked response at the beginning (D1) and after 5–10 days of neutral exposure (Exposed), we found no significant difference in the peak amplitude across the recorded areas, except for a slight, but significant, increase of the response in PtA (Wilcoxon signed-rank test, D1 versus Exposed: $p = 0.047$). In particular, the mice that were exposed did not show any development of the sensory-evoked response in mPFC and dCA1 (Wilcoxon signed-rank test, D1 versus Exposed: mPFC, $p = 0.23$; dCA1, $p = 0.74$) (Figures 3A and 3B). Receiver operating characteristic (ROC) analysis on LFP recordings showed similar stimulus detection

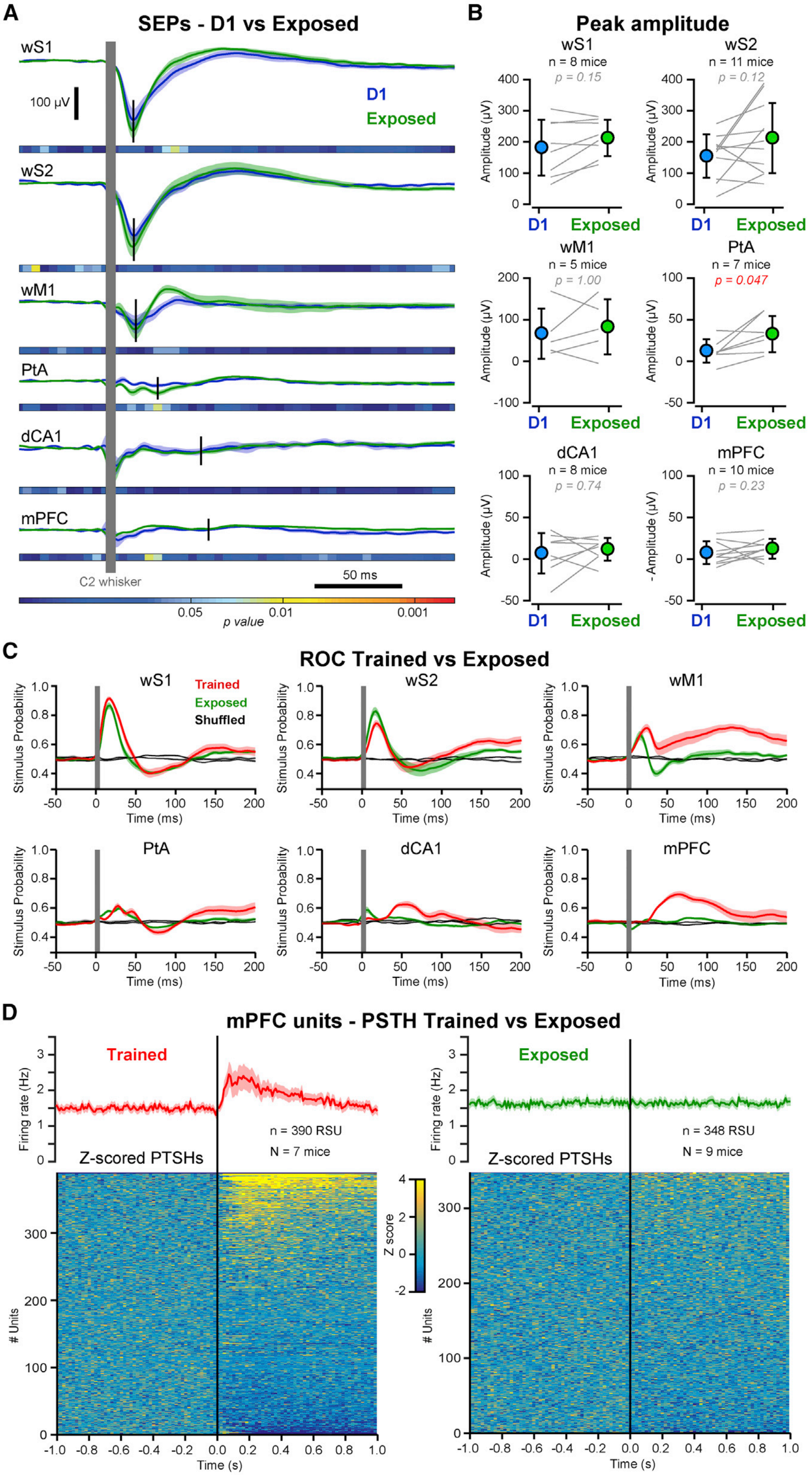

Figure 3. Task-Specific Development of the Sensory-Evoked Responses in dCA1 and mPFC

(A) Grand average SEPs computed for all whisker stimuli, on the first day (D1, blue) and after 5–10 days of neutral exposure (Exposed, green) for the same mice. Shaded areas indicate SEM. Horizontal bars indicate color-coded p values for statistical test of the difference between D1 and Exposed conditions across time (Wilcoxon signed-rank test, D1 versus Exposed for 5 ms time windows). Vertical black bars indicate the times of the peak responses.

(B) Comparison of the peak amplitude of the SEPs for D1 (blue) and Exposed (green) conditions. Circles with bars indicate mean ± SD. Gray lines represent individual mice. The red p value indicates a statistically significant difference between D1 and Exposed (Wilcoxon signed-rank test).

(C) Receiver operating characteristic (ROC) analysis. Average performance (mean ± SEM) of a classifier in decoding the stimulus probability from LFP activity in each area recorded in mice trained in the detection task (Trained, red) and in mice after neutral exposure (Exposed, green). Black lines with gray shading indicate performance of the classifier for label-shuffled distributions.

(D) Top: grand average PSTHs computed for all whisker stimuli from 390 RSUs recorded in the mPFC of mice trained in the detection task (red, N = 7 mice) and from 348 RSUs recorded in the mPFC of mice after neutral exposure (green, N = 9 mice). Shaded areas indicate SEM. Bottom: *Z* scored PSTHs for the RSUs recorded in mice trained in the detection task (left) and the RSUs recorded in mice after neutral exposure (right). PSTHs are sorted according to change in firing rate after whisker stimulus.

probability in sensory areas for mice trained in the detection task or mice after neutral exposure, whereas a significant stimulus detection probability was observed in mPFC and dCA1 only in mice trained in the detection task (Figure 3C), consistent with sensory-evoked responses developing in mPFC and dCA1 only for relevant stimuli. High-density extracellular recordings in mPFC of mice after 10 days of neutral exposure confirmed the absence of any sensory-evoked response in Exposed mice in contrast with the prominent evoked response in mPFC of mice trained in the detection task (Figure 3D).

Having established that whisker sensory responses develop in parallel to task learning over days, we next examined the trial-by-trial correlation between sensory-evoked response and performance in mice trained in the detection task. We compared sensory-evoked responses for Hit and Miss trials. We found that the early sensory-evoked response was smaller for Miss trials compared to Hit trials in most cortical areas, except wS1 (Figures 4A and 4B). The late (100–200 ms) sensory-evoked response was significantly smaller for Miss trials in all areas, including wS1, consistent with previous findings (Sachidhanandam et al., 2013; Yang et al., 2016; Yamashita and Petersen, 2016). Single units in mPFC also showed a strong Hit versus Miss difference with smaller evoked responses in Miss trials, both for positively and negatively modulated RSUs (Figures 4C and S4A). The enhanced sensory processing in Hit trials could therefore contribute to driving task execution.

To address the causal role of the different cortical areas, we next carried out local inactivation experiments in mice trained in the detection task. In a first set of experiments, we trained VGAT-ChR2 mice expressing ChR2 in GABAergic neurons (Zhao et al., 2011) in the detection task. Once they reached good performance (Hit rate = 0.85 ± 0.10; False-Alarm rate = 0.21 ± 15), we performed focal unilateral photo-inactivation. Blue laser pulse trains (200 Hz, 100 ms before to 500 ms after trial start) were applied unilaterally through an optic fiber (300–400 μm in diameter) to one of the six cortical areas in one-third of the trials (both Stimulus trials and Catch trials) (Figure 4D). Focal optogenetic inhibition of wS1 and wS2, but not wM1, induced a marked decrease in Hit rate (Wilcoxon signed-rank test, No-light versus Light trials: wS1, $p = 0.004$; wS2, $p = 0.016$; wM1, $p = 0.31$). In higher-order areas, inhibition of PtA had no effect on Hit rate but significantly increased False-Alarm rate (Wilcoxon signed-rank test, No-light versus Light trials: Hit, $p = 0.46$; False-Alarm, $p = 0.039$). In contrast, unilateral inhibition of dCA1 or mPFC produced a strong reduction in Hit rate (Wilcoxon signed-rank test, No-light versus Light trials: dCA1, $p = 0.016$; mPFC, $p = 0.016$) without affecting False-Alarm rate. Similar results were obtained in another set of experiments using local pharmacological inactivation with muscimol (a GABAergic agonist) in wild-type mice (Figure S4B). Thus, neural activity in both sensory areas (wS1 and wS2) and higher-order areas (dCA1 and mPFC) appears to be required on the millisecond timescale for task execution.

DISCUSSION

Learning, context, attention, and motivation play important roles in the processing of relevant sensory information, and many brain regions likely contribute. Here, we found that both mPFC and dCA1 were specifically recruited during learning of a whisker detection task, showing fast activity that correlated with performance and was required for task execution.

The early sensory responses in wS1 and wS2 were only weakly modulated by detection task learning (Figure 2) or neutral exposure (Figure 3), showing only small differences comparing Hit and Miss trials (Figure 4). These LFP data are consistent with the small Hit versus Miss differences in the early sensory response occurring within the first 50 ms after whisker deflection during closely related detection tasks measured previously using whole-cell electrophysiological recordings from wS1 (Sachidhanandam et al., 2013; Yang et al., 2016; Yamashita and Petersen, 2016) and voltage-sensitive dye imaging experiments (Kyriakatos et al., 2017). At later times (>50 ms), however, reverberatory activities between wS1 and wS2 or with higher-order areas (Manita et al., 2015; Kwon et al., 2016) may contribute significantly to context-dependent sensory perception and task performance (Sachidhanandam et al., 2013; Yang et al., 2016). Moreover, we recently found that the late sensory-evoked response in wS2-projecting neurons in wS1 correlates strongly with performance and developed through learning (Yamashita and Petersen, 2016). Because LFP measures large-scale synaptic signaling, our data do not rule out changes in specific subsets of neurons or synapses in wS1/wS2, which could contribute to task learning and execution (Kwon et al., 2016). Inactivation of wS1/wS2 impaired detection task performance (Figure 4) in agreement with previous studies of whisker detection tasks (Miyashita and Feldman, 2013; Sachidhanandam et al., 2013; Yang et al., 2016; Kwon et al., 2016). wS1/wS2 project to various other brain regions that might contribute to task execution, including frontal cortex (Guo et al., 2014), striatum (Sippy et al., 2015), thalamus, superior colliculus, and brainstem (Aronoff et al., 2010). Overall, our data support the hypothesis that whisker sensory signals need to transit through wS1/wS2 to initiate the goal-directed sensorimotor transformation underlying detection task performance.

Whisker-deflection-evoked responses developed selectively during learning of the detection task in mPFC, but not during neutral exposure (Figures 2 and 3). Both LFP and single units in mPFC of mice trained in the detection task responded rapidly to whisker deflection with latencies well below 50 ms, thus occurring before onset of EMG activity and licking (Figures 1 and S3). On a trial-by-trial basis, responses in mPFC were larger in Hit trials compared to Miss trials (Figure 4). Inactivation of mPFC caused a strong impairment of task performance (Figure 4). Our data are consistent with recent cellular imaging studies in the prelimbic area of mPFC showing neuronal activity evoked by reward-predictive sensory cues in trained mice, whereas little or no activity was evoked by irrelevant sensory stimuli (Pinto and Dan, 2015) or in naive mice (Otis et al., 2017). Fast, learned responses in mPFC may thus contribute on the millisecond timescale to the conversion of sensory signals into goal-directed motor output. The specific development of sensory responses during learning in mPFC may involve ascending neuromodulators, such as dopamine or acetylcholine, signaling the behavioral relevance of the sensory stimulus

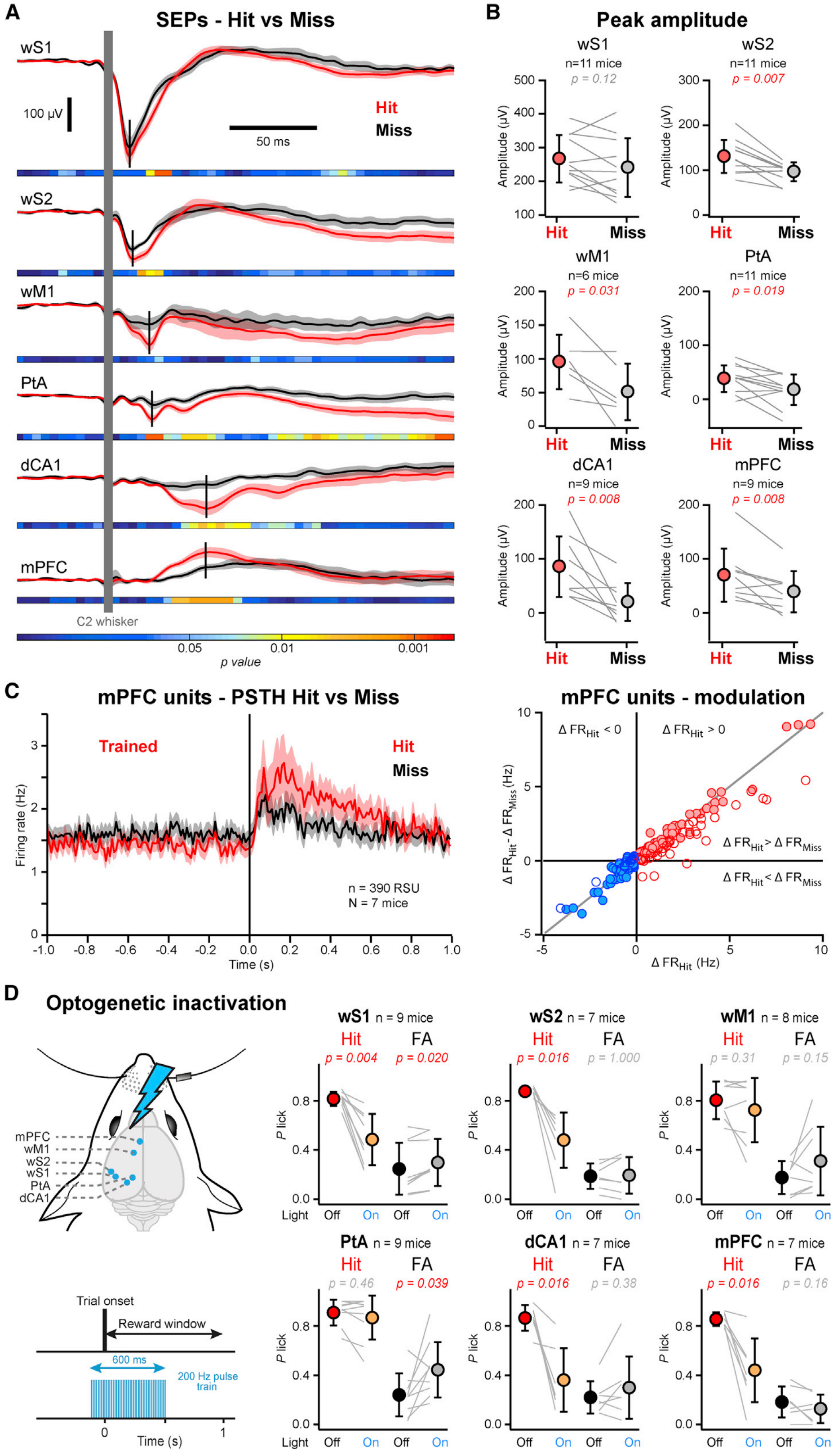

(legend on next page)

(Gritton et al., 2016; Popescu et al., 2016; Teles-Grilo Ruivo et al., 2017).

LFP measurements also revealed a sensory response in dCA1 with a short latency below 50 ms (Figure 1), which increased in parallel with task learning (Figure 2) and was absent in neutral exposure mice (Figure 3). The dCA1 response was larger in Hit trials compared to Misses, and inactivation of dCA1 impaired task performance (Figure 4). Thus, learning induces fast sensory processing of relevant sensory stimuli in dCA1, and the activity of these neurons appears to be necessary for task execution. Sensory processing has previously been reported in hippocampus, including whisker-evoked responses (Pereira et al., 2007; Itskov et al., 2011) and frequency-tuned auditory responses (Aronov et al., 2017; Terada et al., 2017). Whisker-related sensory information could reach the hippocampus through projections from wS1/wS2 to the temporal association area and perirhinal cortex (Aronoff et al., 2010), which in turn signal to hippocampal-related brain regions. The hippocampus is thought to contribute prominently to associative learning (Langston et al., 2010; Pennartz et al., 2011) and could thus be involved in contextual, experience-dependent learning of rewarded sensory-motor associations. Reverberant cortico-hippocampal interactions may contribute to reactivation of specific experience-dependent cortical states (Ji and Wilson, 2007; Girardeau and Zugaro 2011). It is thus possible that the learned sensory responses in dCA1 during our whisker detection task might be involved in context-dependent reactivation of the cortical circuits contributing to driving licking motor output.

Interestingly, mPFC and hippocampus have been shown to interact closely in a variety of goal-directed behaviors (Benchenane et al., 2010; Fujisawa and Buzsáki 2011; Euston et al., 2012; Place et al., 2016). The convergence of task-dependent signals from the mPFC and hippocampus in the nucleus accumbens (Goto and Grace, 2005; Pennartz et al., 2011)—an area critically involved in reward-guided behaviors (Goto and Grace, 2005; Pennartz et al., 2011; Howe and Dombeck, 2016)—could play an important role in the selection and triggering of the appropriate motor command.

Altogether, our study demonstrates a fast and differential recruitment of sensory and high-order cortical areas. Whereas sensory areas responded regardless of the context, processing in dCA1 and mPFC depended upon learning. In future studies, it will be important to identify the circuits and synaptic mechanisms leading to the task-dependent development of sensory-evoked responses in mPFC and dCA1 and to determine the downstream circuits responsible for selecting and triggering the appropriate motor output. In particular, it would be important to determine how sensory information ultimately reaches brain areas driving the behavioral response, such as the striatum (Sippy et al., 2015) and motor-related frontal cortical areas (Guo et al., 2014; Li et al., 2015).

STAR★METHODS

Detailed methods are provided in the online version of this paper and include the following:

- KEY RESOURCES TABLE
- CONTACT FOR REAGENT AND RESOURCE SHARING
- EXPERIMENTAL MODEL AND SUBJECT DETAILS
- METHOD DETAILS
  - Surgery
  - Behavior
  - Multisite local field potential recordings
  - Silicon probe recordings
  - Pharmacological inactivation
  - Optogenetic inactivation
- QUANTIFICATION AND STATISTICAL ANALYSIS
  - Behavior quantification and selection of trained days
  - LFP database and analysis
  - Spike sorting and analysis
  - Receiver Operating Characteristic (ROC) Analysis
  - Statistical analysis
- DATA AND SOFTWARE AVAILABILITY

SUPPLEMENTAL INFORMATION

Supplemental Information includes four figures and can be found with this article online at https://doi.org/10.1016/j.neuron.2017.11.031.

ACKNOWLEDGMENTS

We thank L. Fernandez, M. Perier, and A. Oryshchuk for technical assistance and help with the experiments. This work was supported by INSERM and University Claude Bernard – Lyon1 and funded by grants from Agence Nationale de la Recherche (Project AWAKE CX, ANR-09-JCJC-0028-01, 2009), Lyon Neuroscience Research Center (Projet Structurant, 2009), the Swiss National

Figure 4. Neuronal Activity in mPFC and dCA1 Causally Contributes to Execution of the Detection Task

(A) Grand average SEPs computed for Hit (red) and Miss (black) trials from mice trained in the detection task. Shaded areas indicate SEM. Horizontal bars indicate color-coded p values for statistical test of the difference between Hit and Miss trials across time (Wilcoxon signed-rank test, Hit versus Miss for 5 ms time windows). Vertical black bars indicate the times of the peak responses.

(B) Comparison of the peak amplitude of the SEPs for Hit (red) and Miss (gray) trials. Circles with bars indicate mean ± SD. Gray lines represent individual mice. Red p values indicate statistically significant differences between Hit and Miss (Wilcoxon signed-rank test).

(C) Left: grand average PSTHs (mean ± SEM) computed for Hit (red) and Miss (black) trials from 390 RSUs recorded in the mPFC of mice trained in the detection task (N = 7 mice). Right: scatterplot of the Hit-Miss difference in firing rate against the change in firing rate relative to prestimulus baseline in Hit trials for all significantly modulated mPFC RSUs recorded in trained mice. Red and blue circles represent positively and negatively modulated units, respectively. Filled circles indicate significant Hit-Miss difference.

(D) Left: schematic drawing of the sites targeted for optogenetic inactivation on the contralateral hemisphere to the stimulated whisker in VGAT-ChR2 mice during the detection task. Right: comparison of Hit rates (red and orange) and False-Alarm rates (black and gray) computed for trials with (Light On) or without (Light Off) photo-inhibition applied to wS1, wS2, wM1, PtA, dCA1, or mPFC. Closed circles with error bars indicate mean ± SD. Gray lines indicate individual mice. Red p values indicate statistically significant differences between control trials and trials with photo-inhibition (Wilcoxon signed-rank test).

Science Foundation (310030B_166595), and the European Research Council (ERC-2011-ADG 293660).

AUTHOR CONTRIBUTIONS

Conceptualization, P.L.M., V.E., P.-A.S., C.C.H.P., and S.C.; Methodology, P.L.M. and V.E.; Software, P.L.M. and V.E.; Formal Analysis, P.L.M. and V.E.; Investigation, P.L.M., V.E., E.C., K.G., and S.C.; Writing – Original Draft, C.C.H.P. and S.C.; Writing – Review & Editing, P.L.M., V.E., P.-A.S., C.C.H.P., and S.C.; Visualization, P.L.M., C.C.H.P., and S.C.; Supervision, P.-A.S., C.C.H.P., and S.C.; Funding Acquisition, P.-A.S., C.C.H.P., and S.C.

Received: September 29, 2017
Revised: November 10, 2017
Accepted: November 17, 2017
Published: December 14, 2017

REFERENCES

Aronoff, R., Matyas, F., Mateo, C., Ciron, C., Schneider, B., and Petersen, C.C.H. (2010). Long-range connectivity of mouse primary somatosensory barrel cortex. Eur. J. Neurosci. *31*, 2221–2233.

Aronov, D., Nevers, R., and Tank, D.W. (2017). Mapping of a non-spatial dimension by the hippocampal-entorhinal circuit. Nature *543*, 719–722.

Benchenane, K., Peyrache, A., Khamassi, M., Tierney, P.L., Gioanni, Y., Battaglia, F.P., and Wiener, S.I. (2010). Coherent theta oscillations and reorganization of spike timing in the hippocampal- prefrontal network upon learning. Neuron *66*, 921–936.

Buzsáki, G. (2004). Large-scale recording of neuronal ensembles. Nat. Neurosci. *7*, 446–451.

Euston, D.R., Gruber, A.J., and McNaughton, B.L. (2012). The role of medial prefrontal cortex in memory and decision making. Neuron *76*, 1057–1070.

Ferezou, I., Haiss, F., Gentet, L.J., Aronoff, R., Weber, B., and Petersen, C.C.H. (2007). Spatiotemporal dynamics of cortical sensorimotor integration in behaving mice. Neuron *56*, 907–923.

Fernandez, L.M., Comte, J.C., Le Merre, P., Lin, J.S., Salin, P.A., and Crochet, S. (2017). Highly dynamic spatiotemporal organization of low-frequency activities during behavioral states in the mouse cerebral cortex. Cereb. Cortex *27*, 5444–5462.

Fujisawa, S., and Buzsáki, G. (2011). A 4 Hz oscillation adaptively synchronizes prefrontal, VTA, and hippocampal activities. Neuron *72*, 153–165.

Girardeau, G., and Zugaro, M. (2011). Hippocampal ripples and memory consolidation. Curr. Opin. Neurobiol. *21*, 452–459.

Goto, Y., and Grace, A.A. (2005). Dopaminergic modulation of limbic and cortical drive of nucleus accumbens in goal-directed behavior. Nat. Neurosci. *8*, 805–812.

Gritton, H.J., Howe, W.M., Mallory, C.S., Hetrick, V.L., Berke, J.D., and Sarter, M. (2016). Cortical cholinergic signaling controls the detection of cues. Proc. Natl. Acad. Sci. USA *113*, E1089–E1097.

Guo, Z.V., Li, N., Huber, D., Ophir, E., Gutnisky, D., Ting, J.T., Feng, G., and Svoboda, K. (2014). Flow of cortical activity underlying a tactile decision in mice. Neuron *81*, 179–194.

Harvey, C.D., Coen, P., and Tank, D.W. (2012). Choice-specific sequences in parietal cortex during a virtual-navigation decision task. Nature *484*, 62–68.

Hautus, M.J. (1995). Corrections for extreme proportions and their biasing effects on estimated values of d’. Behav. Res. Meth. Instrum. *27*, 46–51.

Howe, M.W., and Dombeck, D.A. (2016). Rapid signalling in distinct dopaminergic axons during locomotion and reward. Nature *535*, 505–510.

Itskov, P.M., Vinnik, E., and Diamond, M.E. (2011). Hippocampal representation of touch-guided behavior in rats: persistent and independent traces of stimulus and reward location. PLoS ONE *6*, e16462.

Ji, D., and Wilson, M.A. (2007). Coordinated memory replay in the visual cortex and hippocampus during sleep. Nat. Neurosci. *10*, 100–107.

Kwon, S.E., Yang, H., Minamisawa, G., and O’Connor, D.H. (2016). Sensory and decision-related activity propagate in a cortical feedback loop during touch perception. Nat. Neurosci. *19*, 1243–1249.

Kyriakatos, A., Sadashivaiah, V., Zhang, Y., Motta, A., Auffret, M., and Petersen, C.C.H. (2017). Voltage-sensitive dye imaging of mouse neocortex during a whisker detection task. Neurophotonics *4*, 031204.

Langston, R.F., Stevenson, C.H., Wilson, C.L., Saunders, I., and Wood, E.R. (2010). The role of hippocampal subregions in memory for stimulus associations. Behav. Brain Res. *215*, 275–291.

Li, N., Chen, T.W., Guo, Z.V., Gerfen, C.R., and Svoboda, K. (2015). A motor cortex circuit for motor planning and movement. Nature *519*, 51–56.

Manita, S., Suzuki, T., Homma, C., Matsumoto, T., Odagawa, M., Yamada, K., Ota, K., Matsubara, C., Inutsuka, A., Sato, M., et al. (2015). A top-down cortical circuit for accurate sensory perception. Neuron *86*, 1304–1316.

Miyashita, T., and Feldman, D.E. (2013). Behavioral detection of passive whisker stimuli requires somatosensory cortex. Cereb. Cortex *23*, 1655–1662.

Mohajerani, M.H., Chan, A.W., Mohsenvand, M., LeDue, J., Liu, R., McVea, D.A., Boyd, J.D., Wang, Y.T., Reimers, M., and Murphy, T.H. (2013). Spontaneous cortical activity alternates between motifs defined by regional axonal projections. Nat. Neurosci. *16*, 1426–1435.

Otis, J.M., Namboodiri, V.M., Matan, A.M., Voets, E.S., Mohorn, E.P., Kosyk, O., McHenry, J.A., Robinson, J.E., Resendez, S.L., Rossi, M.A., and Stuber, G.D. (2017). Prefrontal cortex output circuits guide reward seeking through divergent cue encoding. Nature *543*, 103–107.

Paxinos, G., and Franklin, K.B.J. (2008). The Mouse Brain in Stereotaxic Coordinates, Third Edition (Elsevier).

Pennartz, C.M., Ito, R., Verschure, P.F., Battaglia, F.P., and Robbins, T.W. (2011). The hippocampal-striatal axis in learning, prediction and goal-directed behavior. Trends Neurosci. *34*, 548–559.

Pereira, A., Ribeiro, S., Wiest, M., Moore, L.C., Pantoja, J., Lin, S.C., and Nicolelis, M.A. (2007). Processing of tactile information by the hippocampus. Proc. Natl. Acad. Sci. USA *104*, 18286–18291.

Pinto, L., and Dan, Y. (2015). Cell-type-specific activity in prefrontal cortex during goal-directed behavior. Neuron *87*, 437–450.

Place, R., Farovik, A., Brockmann, M., and Eichenbaum, H. (2016). Bidirectional prefrontal-hippocampal interactions support context-guided memory. Nat. Neurosci. *19*, 992–994.

Popescu, A.T., Zhou, M.R., and Poo, M.M. (2016). Phasic dopamine release in the medial prefrontal cortex enhances stimulus discrimination. Proc. Natl. Acad. Sci. USA *113*, E3169–E3176.

Romo, R., and de Lafuente, V. (2013). Conversion of sensory signals into perceptual decisions. Prog. Neurobiol. *103*, 41–75.

Rossant, C., Kadir, S.N., Goodman, D.F.M., Schulman, J., Hunter, M.L.D., Saleem, A.B., Grosmark, A., Belluscio, M., Denfield, G.H., Ecker, A.S., et al. (2016). Spike sorting for large, dense electrode arrays. Nat. Neurosci. *19*, 634–641.

Sachidhanandam, S., Sreenivasan, V., Kyriakatos, A., Kremer, Y., and Petersen, C.C.H. (2013). Membrane potential correlates of sensory perception in mouse barrel cortex. Nat. Neurosci. *16*, 1671–1677.

Siegel, M., Buschman, T.J., and Miller, E.K. (2015). Cortical information flow during flexible sensorimotor decisions. Science *348*, 1352–1355.

Sippy, T., Lapray, D., Crochet, S., and Petersen, C.C.H. (2015). Cell-type-specific sensorimotor processing in striatal projection neurons during goal-directed behavior. Neuron *88*, 298–305.

Song, Y.H., Kim, J.H., Jeong, H.W., Choi, I., Jeong, D., Kim, K., and Lee, S.H. (2017). A neural circuit for auditory dominance over visual perception. Neuron *93*, 940–954.e6.

Teles-Grilo Ruivo, L.M., Baker, K.L., Conway, M.W., Kinsley, P.J., Gilmour, G., Phillips, K.G., Isaac, J.T.R., Lowry, J.P., and Mellor, J.R. (2017). Coordinated

acetylcholine release in prefrontal cortex and hippocampus is associated with arousal and reward on distinct timescales. Cell Rep. *18*, 905–917.

Terada, S., Sakurai, Y., Nakahara, H., and Fujisawa, S. (2017). Temporal and rate coding for discrete event sequences in the hippocampus. Neuron *94*, 1248–1262.e4.

Yamashita, T., and Petersen, C.C.H. (2016). Target-specific membrane potential dynamics of neocortical projection neurons during goal-directed behavior. eLife *5*, e15798.

Yang, H., Kwon, S.E., Severson, K.S., and O'Connor, D.H. (2016). Origins of choice-related activity in mouse somatosensory cortex. Nat. Neurosci. *19*, 127–134.

Zhao, S., Ting, J.T., Atallah, H.E., Qiu, L., Tan, J., Gloss, B., Augustine, G.J., Deisseroth, K., Luo, M., Graybiel, A.M., and Feng, G. (2011). Cell type–specific channelrhodopsin-2 transgenic mice for optogenetic dissection of neural circuitry function. Nat. Methods *8*, 745–752.

STAR★METHODS

KEY RESOURCES TABLE

REAGENT or RESOURCE	SOURCE	IDENTIFIER
Chemicals, Peptides, and Recombinant Proteins		
Muscimol [2763-96-4]	Biotrend USA	Cat# BN0352
DiI Stain (1,1'-Dioctadecyl-3,3,3′,3′-Tetramethylindocarbocyanine Perchlorate)	Invitrogen	Cat# D282
Experimental Models: Organisms/Strains		
Mice C57BL/6	Janvier labs	C57BL/6J
Mice VGAT-ChR2-EYFP	The Jackson Laboratory	Jax014548; RRID: IMSR_JAX:014548
Software and Algorithms		
Labview	National Instruments	http://www.ni.com/en-us.html
Klusta	https://github.com/kwikteam/klusta	Rossant et al., 2016
MATLAB R2015b	MathWorks	https://www.mathworks.com/
Other		
High-impedance LFP electrodes	FHC	UEWSCGSELNND
Extracellular amplifier for LFP recording	A-M Systems	Model 3000 AC/DC Differential Amplifier (Custom modified)
Vision XP	Nicolet	https://www.hbm.com/en/
Silicon probes A32-poly2	Neuronexus	A1x32-Poly2-10mm-50 s-177
Extracellular multichannel amplifier for silicon probe recording	Blackrock Microsystems	CerePlex Direct
Blue (473 nm) DPSS laser with TEM00 mode, high power	GMP	http://www.gmp.ch/
Chronic LFP database and analysis codes	This paper	https://doi.org/10.5281/zenodo.1063898
Acute mPFC spike recordings and analysis codes	This paper	https://doi.org/10.5281/zenodo.1063898
Optogenetic inactivation and analysis codes	This paper	https://doi.org/10.5281/zenodo.1063898
Muscimol inactivation and analysis codes	This paper	https://doi.org/10.5281/zenodo.1063898

CONTACT FOR REAGENT AND RESOURCE SHARING

Further information and requests for resources and reagents should be directed to and will be fulfilled by the Lead Contact, Sylvain Crochet (sylvain.crochet@epfl.ch).

EXPERIMENTAL MODEL AND SUBJECT DETAILS

All procedures were approved by the University of Lyon 1 Animal Care Committee (project DR2013-47) and Swiss Federal Veterinary Office (License number 1628) and were conducted in accordance with the French, Swiss and European Community guidelines for the use of research animals. All efforts were made to minimize the number of animals used and their suffering. Twenty-six adult male C57BL/6 mice (Janvier SAS, St. Berthevin, France; 6-8 week old at the time of surgery) were used for chronic local field potential (LFP) recordings during the detection task (n = 14 mice) or the neutral exposure (n = 12 mice). Sixteen adult male C57BL/6 mice were used for acute extracellular recordings using silicon probes during the detection task or neutral exposure. Thirty-three adult male and female C57BL/6 mice were used for acute pharmacological inactivation and control experiments during the detection task. Nineteen adult male and female VGAT-ChR2-EYFP transgenic mice (Jackson Laboratory, Jax014548) were used for local optogenetic inactivation during the detection task. The mice implanted for LFP recording were housed individually to avoid deterioration of the implanted connector. For other experiments, the mice were housed in groups of 2-4 mice. Mice were kept in a reverse

light/dark cycle (light 7 p.m. to 7 a.m.), at a temperature of 22 ± 2°C with food available *ad libitum*. Water was restricted to 1 ml a day during behavioral training with at least 2 days of free-access to water in the cage every 2 weeks. All mice were weighed and inspected daily during behavioral training.

METHOD DETAILS

Surgery

For chronic local field potential (LFP) recordings

Adult mice (6-8 weeks) were anesthetized with isoflurane supplemented with a mixture of N_2O and O_2. Carprofen (s.c., 5 mg/kg) was administered before the surgery. Subcutaneous injections of saline (0.10-0.15 ml NaCl 0.9%) were administered every hour during the surgery to prevent dehydration. A heating blanket maintained the rectally measured body temperature at 37°C. The head of the mouse was fixed in a stereotaxic apparatus (Kopf) using ear-bars. An ocular ointment (Viscotears, Alcon) was applied over the eyes to prevent drying. A mixture of Lidocaine 2% and Bupivacaine 0.5% was injected locally before skin incision. The skin overlying the cortex was removed and the bone gently cleaned. The periosteal tissue, covering the scalp, was removed by gently scraping with a scalpel blade. A thin layer of glue was applied on the exposed skull. Six high-impedance sharp tungsten microelectrodes (10-12 MOhm, 75 μm shaft diameter, from FHC) were stereotaxically implanted individually using interaural coordinates (Paxinos and Franklin 2008) (Figure S1A). The recording sites included: the barrel field of the primary somatosensory cortex (wS1: AP 1.95 mm; Lat 3.5 mm; Depth from surface 0.5 mm); the whisker secondary somatosensory cortex (wS2: AP 2.1 mm; Lat 4.2 mm; Depth from surface 0.5 mm); the whisker primary motor area (wM1: AP 4.8 mm; Lat 1.0 mm; Depth from surface 0.4 mm); the parietal associative area (PtA: AP 1.85 mm; Lat 1.6 mm; Depth from surface 0.5 mm); the prelimbic area of the medial prefrontal cortex (mPFC: AP 5.8 mm; Lat 0.3 mm; Depth from surface 1.85 mm) and the CA1 area of the dorsal hippocampus (dCA1: AP 1.3 mm; Lat 2.0 mm; Depth from surface 1.3 mm). Small craniotomies (~300 μm in diameter) were performed to allow the insertion of each electrode that was slowly lowered vertically to the recording depth. For neocortical areas, the tip of the electrode was lowered to a depth of 300-400 μm from the pia. For the hippocampus, we targeted the stratum radiatum of dCA1. When in position the electrodes were glued to the skull (Cyanoacrylate adhesive, Sigma Aldrich) and cemented using acrylate dental cement (Palavit). Each electrode was then soldered to a small electric connector. In some mice, two conventional surface EEG electrodes were implanted onto the duramater over the parietal (AP 2.0, Lat 1.5) and frontal areas (AP 5.3, Lat 1.5) of the contralateral hemisphere. Two electrodes were inserted in the neck muscles for nuchal EMG recordings. Two silver wires were inserted in contact with the cerebellum on both sides for reference and grounding. A light-weight metal head-post was also cemented to the skull allowing painless head-fixation during recording sessions. After the surgery, the animal was returned to its home cage and the analgesic Ibuprofen was added to the drinking water for 3 days following surgery. At the end of the recording sessions, the animals were deeply anesthetized with pentobarbital (60 mg/kg; i.p.). Small electrolytic lesions were performed to localize the position of each electrode (Figure S1B). The animals were then transcardially perfused with 4% paraformaldehyde (PFA). The brain was removed and post-fixed in 4% PFA. Brain sections of 100 μm were cut to identify the recording sites.

For acute inactivation and extracellular recording experiments

Adult mice (5-8 weeks) were anesthetized with a mixture of ketamine and xylazine (ketamine: 125 mg/kg, xylazine 10 mg/kg, i.p.). Carprofen (0.5 mg/ml, 300 μl, i.p) was administered before the surgery. The body temperature was maintained at 37°C by a heating pad. An ocular ointment (Viscotears, Alcon) was applied over the eyes. The head of the mouse was fixed in a stereotaxic apparatus (Kopf). A mixture of Lidocaine 2% and Bupivacaine 0.5% was injected locally before skin incision. The skin overlying the cortex was removed, the skull was cleaned with Betadine and the bone gently cleaned. A thin layer of glue was applied on the exposed skull. A light-weight metal head-post was fixed to the right hemisphere with cyano-acrylate glue (Henkel, Dusseldorf, Germany) and dental cement (Paladur, Heraeus Kulzer, Hanau, Germany). A chamber was made by building a wall with dental cement along the edge of the bone covering the left hemisphere. Targeted cortical areas for inactivation were marked using stereotaxic coordinates on the surface of the skull, except for wS1 and wS2 that were targeted using intrinsic optical imaging. After the surgery, the animal was returned to its home cage and the analgesic Ibuprofen was added to the drinking water for 3 days following surgery.

Optogenetic inactivation of superficial cortical areas (wS1, wS2, wM1 and PtA) was performed through the bone. The bone was thinned just above the targeted area and then covered with a thin layer of glue under isoflurane anesthesia the day before. For pharmacological inactivation, optogenetic inactivation of deep cortical areas (mPFC and dCA1) and acute mPFC recording with silicon probes, a small craniotomy (300-500 μm in diameter) was opened a few hours (> 3 h) or the day before the experiment to access the targeted cortical area. The mice were anesthetized with isoflurane (3% for induction then 1%–2%). Carprofen (0.5 mg/ml, 300 μl, i.p) was administered before the surgery. The open craniotomy was covered with Silicone sealant (Kwik-Cast, WPI) and the mouse was returned to its home cage for recovery.

At the end of the experiments, each mouse was deeply anesthetized with pentobarbital (60 mg/kg; i.p.), and then transcardially perfused with 4% paraformaldehyde (PFA). The brain was removed and post-fixed in 4% PFA. Brain sections of 100 μm were cut to identify injection site, optic fiber position or recording site in mPFC.

Behavior

A total of 72 mice (male and female; wild-type C57BL/6J and VGAT-ChR2-EYFP) were trained in the detection task. Mice were first habituated to be head-restrained over a period of 2-3 days (Figure S2A). The day before training, all whiskers were trimmed except for the C2 whiskers on both sides, and the mice were water restricted to 1 ml of water/day. Their weight and general health status were then carefully monitored every day using a score sheet. Mice were trained daily with one session/day. The training started with two sessions of 'free-licking' during which the mice were rewarded (5 μl of water) every time they licked the water spout if the lick was preceded by a 3-4 s period without any lick (No-lick). Licks were detected with piezo film attached to the reward spout (Figure S2A). The subsequent days, the mice where engaged in one of the two behavioral tasks (detection task or neutral exposure). For both tasks, the right C2 whisker was stimulated using a brief 1 ms magnetic pulse to elicit a vertical deflection that was transmitted by a small metal particle glued on the whisker (Figure S2A) (Sachidhanandam et al., 2013; Sippy et al., 2015; Yamashita and Petersen, 2016). Ambient white noise (80 dB) was played continuously to mask any potential auditory cue generated by the magnetic pulse or external noise that could distract the mice. Behavioral control and behavioral data collection were carried out with custom-written computer routines using a National Instruments board interfaced through LabView or MATLAB (MathWorks).

For the detection task (Figure S2B), trials with whisker stimulation (Stimulus trials) or those without whisker stimulation (Catch trials) were started without any preceding cues, at random inter-trial intervals ranging from 6 to 12 s. Catch trials were randomly interleaved with Stimulus trials, with 50% probability of all trials. If the mouse licked in the 3-4 s no-lick window preceding the time when the trial was supposed to occur, then the trial was aborted (Figure S2B). Catch trials were present from the first day of training. Mice were rewarded only if they licked the water spout within a 1 s response window following the whisker stimulation (Hit). After each training session, the amount of water collected by the mice was computed from the number of Hit trials (Number of Hit x Reward Size) and a supplement of water was given to each mouse to reach a daily amount of 1 ml. The body weight maintained over 80% of the initial value (just before water restriction).

For the neutral exposure task (Figure S2B), 21 mice were trained to collect the reward by licking the water spout with an inter-trial interval ranging from 6 to 12 s and after a no-lick period of 3-4 s, similar to the detection task. At random times the same 1 ms whisker stimulus was delivered to the C2 whisker with an inter-stimulus interval ranging from 6 to 12 s and a probability of 50%. The whisker stimulus was not correlated to the delivery of the reward, therefore, no association between the stimulus and the delivery of the reward could be made. In this behavioral paradigm, mice were exposed to the whisker stimulus during 7-10 days.

Multisite local field potential recordings

Recordings were performed daily during each behavioral session. Each electrode was connected to the head-stage of the amplifier (custom modified Model 3000 AC/DC Differential Amplifiers, A-M Systems, USA). LFPs were recorded using one of the two silver wires implanted in the cerebellum as reference, the other wire being connected to the ground. EEG and EMG were recorded differentially. Signals were band pass filtered between 0.1-1000 Hz for the LFPs and EEG, and 10-20000 Hz for the EMG. The signals were digitized and recorded at 2 kHz on a Vision XP (LDS Nicolet).

Silicon probe recordings

Extracellular spikes in the mPFC were recorded using silicon probes (A1x32-Poly2-10mm-50 s-177, NeuroNexus, MI, USA) with 32 recording sites along a single shank covering 775 μm in depth. The probe was lowered gradually with a 10° angle relative to vertical, until the tip reached a depth of ~2300 μm under the surface of the pia. The probe was coated with DiI (1,1'-dioctadecyl-3,3,3′3'-tetramethylindocarbocyanine perchlorate, Invitrogen, USA) for post hoc recovery of the recording location (Figure S1C). The signals were filtered between 0.3 Hz and 7.5 kHz and amplified using a digital headstage (CerePlex M32, Blackrock Microsystems, UT, USA). The headstage digitized the data with a sampling frequency of 30 kHz. The digitized signal was transferred to our data acquisition system (CerePlex Direct, Blackrock Microsystems, UT, USA) and stored on an internal HDD of the host PC for offline analysis.

Pharmacological inactivation

Pharmacological inactivations were performed on mice trained in the detection task after reaching good performance (mean Hit rate = 0.73 ± 0.14 and mean False-Alarm rate = 0.20 ± 0.14 the day before, n = 33 mice). A small craniotomy was performed under isoflurane anesthesia, over one of the targeted areas a few hours (3-4 h) or a day before. On the test session, behavioral performance in the detection task was assessed during a 5-min control block. Then, 4 or 5 injections of 100 nL of Muscimol (BioTrend, USA) 5 mM (0.62 μg/μl) dissolved in Ringer's, or Ringer's alone for control experiments, were performed at (1000,) 800, 600, 400 and 200 μm below the pia for wS1, wS2, wM1 and PtA, at 1800, 1600, 1400 and 1200 μm below the pia for dCA1 and at (2100,) 2000, 1900, 1800 and 1700 μm below the pia for mPFC using a hydraulic injection system (Narishige) with a glass micropipette (tip 10 to 20 μm) attached to a micromanipulator. Ringer's solution contained (in mM) 135 NaCl, 5 KCl, 5 4-(2-hydroxyethyl)-1-piperazineethanesulfonic acid (HEPES), 1.8 $CaCl_2$, 1 $MgCl_2$. For some injections, 0.1% Chicago Sky Blue was added to visualize the location of the injection site. Thirty minutes after the last injection, the behavioral performance in the detection task was again assessed. One or two inactivation experiments were carried out per mouse spaced by at least one day of recovery with a normal behavioral session.

Optogenetic inactivation

Optogenetic inactivations were performed in VGAT-ChR2 mice trained in the detection task once reaching good performance (mean Hit rate = 0.85 ± 0.10 and mean False-Alarm rate = 0.21 ± 15 on no-light trials, n = 19 mice). The mice were trained in the detection task with an ambient blue masking light. On the testing day, an optic fiber (400 μm; NA = 0.39, Thorlabs) was positioned in contact to the thinned bone for superficial areas or lowered through the cortex just above the left dCA1 at a depth of 1000 μm below the pia. For mPFC, an optic fiber (300 μm; NA = 0.39, Thorlabs) was lowered into the left hemisphere with an angle of 10° (lateral to medial) at a depth of 1700 μm, just above the prelimbic area of mPFC. The optic fiber was connected to a blue Laser (473 nm, GMP). On light trials, a 200 Hz train of blue light pulses (50% duty cycle; peak power of 25-32 mW) was applied 100 ms before the onset of the trial for a duration of 600 ms. Light trains were randomly applied to 30% of both Stimulus and Catch trials (50%–50% Stimulus/Catch trials probability). One to three cortical areas were tested on the same mouse over different sessions, but only one area was inactivated on a given session and at least one control session without any inactivation was performed between two inactivation sessions.

QUANTIFICATION AND STATISTICAL ANALYSIS

Behavior quantification and selection of trained days

For the detection task, the performance of the animals was assessed by computing the Hit rate (number of Hit trials divided by number of Stimulus trials) and the False-Alarm (FA) rate (number of FA trials divided by number of Catch trials) (Figures S2C and S2D). We also computed the d’ as follows: d’ = Z(Hit rate) − Z(FA rate) where the function Z(p), $p \in [0,1]$, is the inverse of the cumulative distribution function of the Gaussian distribution. The loglinear correction for extreme values of d’ has been used systematically (Hautus, 1995). To analyze the sensory evoked response in trained mice, we selected the first 3 days that fulfilled the following criteria: training day > 4 and d’ > 1 (mean = 7.77 ± 1.33 days; range 5-12 days). For the neutral exposure experiments, we selected the last 3 days of neutral exposure (mean = 8.25 ± 1.22 days; range 5-10 days). For the analysis of Hit trials, trials with premature licks occurring within 100 ms after the whisker stimulus were excluded because they may represent licking by chance – similar to False-Alarms – rather than a response to the whisker stimulus.

For pharmacological inactivation experiments, we computed the performance of the mice within a 5 min block, starting 30 min after the end of the injection in order to homogenize and limit the effect of Muscimol diffusion. We then compared the effect of Muscimol injection in a given area to a control group which received Ringer’s injection in different cortical areas (wS1, n = 2 mice; wS2, n = 2 mice; wM1, n = 5 mice; PtA, n = 2 mice; and mPFC, n = 1 mice). For optogenetic inactivation experiments, we directly compared the performance on randomly intermingled trials with and without blue laser pulses for the same session.

LFP database and analysis

For multisite LFP experiments a database of recordings was made after selection of the recordings based on anatomical verification and the absence of electrical artifacts. From the 14 mice recorded during the detection task, we selected: 11 recordings in wS1, 11 recordings in wS2, 6 recordings in wM1, 11 recordings in PtA, 9 recordings in dCA1 and 10 recordings in mPFC. From the 12 mice recorded during the neutral exposure, we selected: 8 recordings in wS1, 11 recordings in wS2, 5 recordings in wM1, 7 recordings in PtA, 8 recordings in dCA1 and 10 recordings in mPFC.

In order to compute the average sensory evoked potentials, the LFPs were band pass filtered between 0.1 and 100 Hz after removing the stimulus artifact elicited by the magnetic pulse. Sensory evoked potentials were then averaged for the different conditions. A baseline correction (50 ms before the trial onset) was applied to every trial. To compute the peak amplitude of the sensory evoked potentials, we determined for each session the time of the first peak in the average sensory evoked potentials computed for all Stimulus trials in trained or exposed mice. Then the peak was measured at the same time point for the different conditions (Hit versus Miss, Trained versus D1, Exposed versus D1).

Spike sorting and analysis

For silicon probe recordings, the spiking activity on each probe was detected and sorted into different clusters using the KlustaSuite (Rossant et al., 2016). After an automated clustering step, clusters were manually sorted and refined. Only well isolated single units were included in the dataset. The isolated units were classified as Fast-Spiking Units (FSUs, putative inhibitory interneurons) and Regular-Spiking Units (RSUs, putative pyramidal cells) based on the duration of the spike waveforms (peak to return-to-baseline time) (Figure S1D).

Receiver Operating Characteristic (ROC) Analysis

ROC analysis was performed from LFP data using the “perfcurve” function from MATLAB. ROC curves were built by comparing the distribution of the mean amplitude of the LFP on a 10 ms time-window for Stimulus and Catch trials. The area under this curve was then used as the Stimulus Probability (SP) for this time-window. The whole SP curve was computed by sliding the 10 ms time-window by steps of 2.5 ms before and after whisker stimulus onset. The grand-average SP curve was then computed by averaging SP curves across mice. Chance level for Stimulus Probability was assessed by shuffling trial labels (Stimulus and Catch trials) 100 times to obtain a mean shuffled SP curve for each mouse.

Statistical analysis

Most statistical analyses were performed using non-parametric tests. Wilcoxon Signed Rank test was used for comparison between two conditions from the same recording sites and mice. Dunn-Holland-Wolfe test was used to compare the latency and peak-time of the sensory evoked response across recorded areas. Mann-Whitney two sample rank test with Bonferroni-Holm correction was used to compare the effect of Muscimol inactivation with Ringer's injection (control group). Correlation between the amplitude of the sensory evoked response and performance (d') was assessed using Pearson correlation with *t* statistics. For mPFC spike recordings, significantly modulated units were computed by comparing the mean firing rate of each unit across hit trials during the 1 s window after trial onset and the 1 s window before trial onset. The *p* values for positively and negatively modulated units were obtained by bootstrapping the trials 1000 times and comparing the distribution of firing rate differences to zero. Values in the text and figures are expressed as mean ± SD, unless otherwise mentioned.

DATA AND SOFTWARE AVAILABILITY

The full dataset and analysis code are available on the CERN Zenodo database: https://doi.org/10.5281/zenodo.1063898.

Neuron, Volume *97*

Supplemental Information

Reward-Based Learning Drives Rapid Sensory Signals in Medial Prefrontal Cortex and Dorsal Hippocampus Necessary for Goal-Directed Behavior

Pierre Le Merre, Vahid Esmaeili, Eloïse Charrière, Katia Galan, Paul-A. Salin, Carl C.H. Petersen, and Sylvain Crochet

Reward-based learning drives rapid sensory signals in medial prefrontal cortex and dorsal hippocampus necessary for goal-directed behavior

Pierre Le Merre, Vahid Esmaeili, Eloïse Charière, Katia Galan, Paul-A. Salin, Carl C.H. Petersen and Sylvain Crochet

Supplemental Information consists of:

Figure S1, related to Figure 1
Figure S2, related to Figure 1
Figure S3, related to Figure 1
Figure S4, related to Figure 4

Figure S1

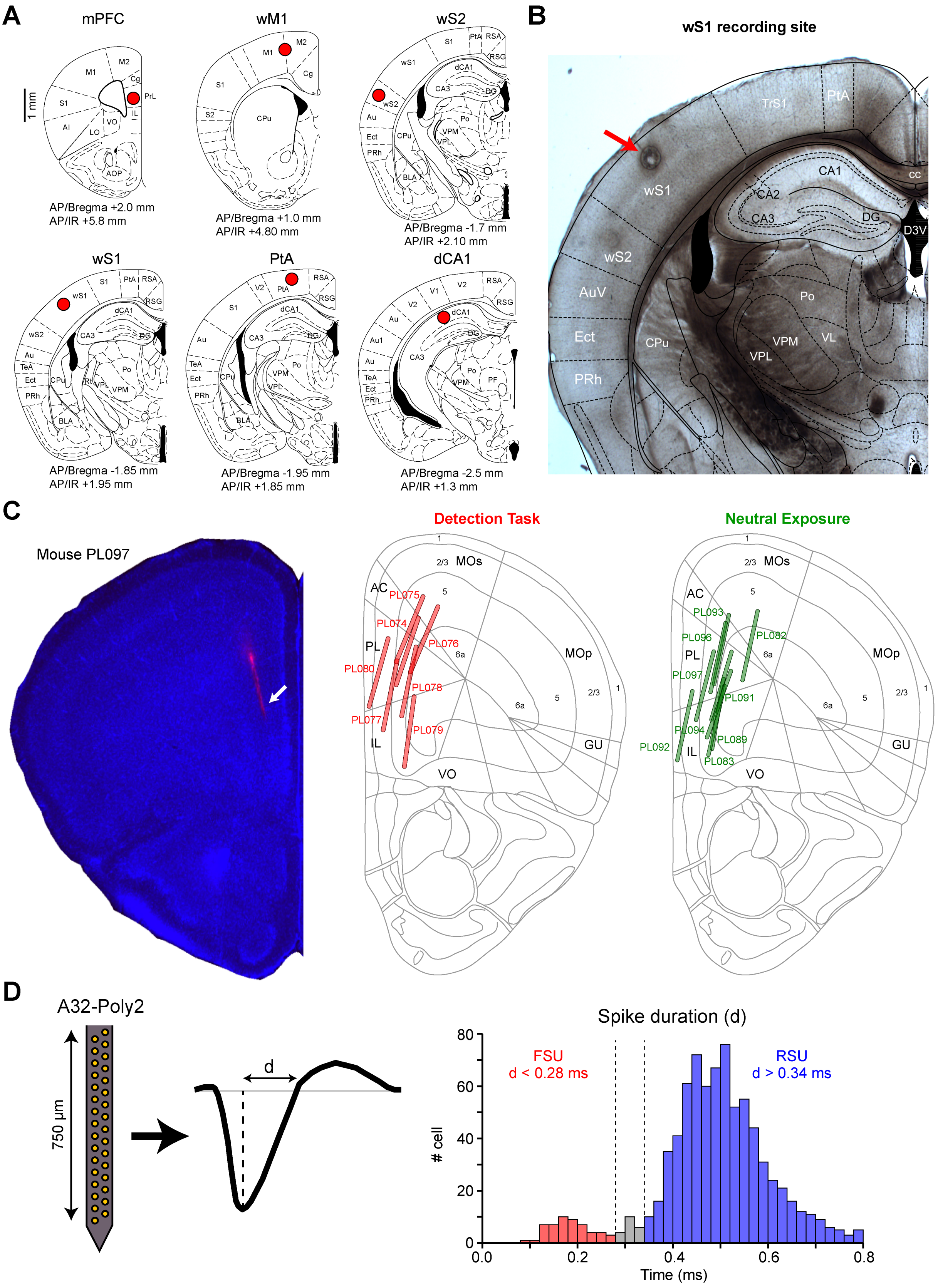

Figure S1. LFP and high-density extracellular recordings

(A) Schematic representation of the targeted cortical areas for multisite LFP recordings. From anterior to posterior: mPFC, wM1, wS2, wS1, PtA and dCA1. Anterio-posterior

coordinates relative to Bregma (AP/Bregma) and interaural (AP/IR) are indicated below each site.

(B) Example anatomical verification of a wS1 LFP recording site. At the end of the experiment, electrolytic lesions were performed under anesthesia for each LFP electrode, before perfusion. 100 µm thick sections were cut to identify recording sites.

(C) *Left*, Example anatomical verification of a silicon-probe recording site in mPFC. The probe was coated with DiI. At the end of the experiment the mouse was perfused. 100 µm thick sections were cut to identify recording sites. *Right*, Location of the silicon-probes in the mPFC of Trained (red) and Exposed (green) mice.

(D) *Left*, Schematic representation of the A32-Poly2 Neuronexus probe used for mPFC extracellular recordings and spike waveform. The duration of the extracellular action-potential (d) was computed as the time between the peak and return-to-baseline. *Right*, Distribution of spike duration for all mPFC recorded units. Units with spike duration below 0.28 ms were classified as fast-spiking units (FSUs, red). Units with spike duration above 0.34 ms were classified as regular-spiking units (RSUs, blue).

Figure S2

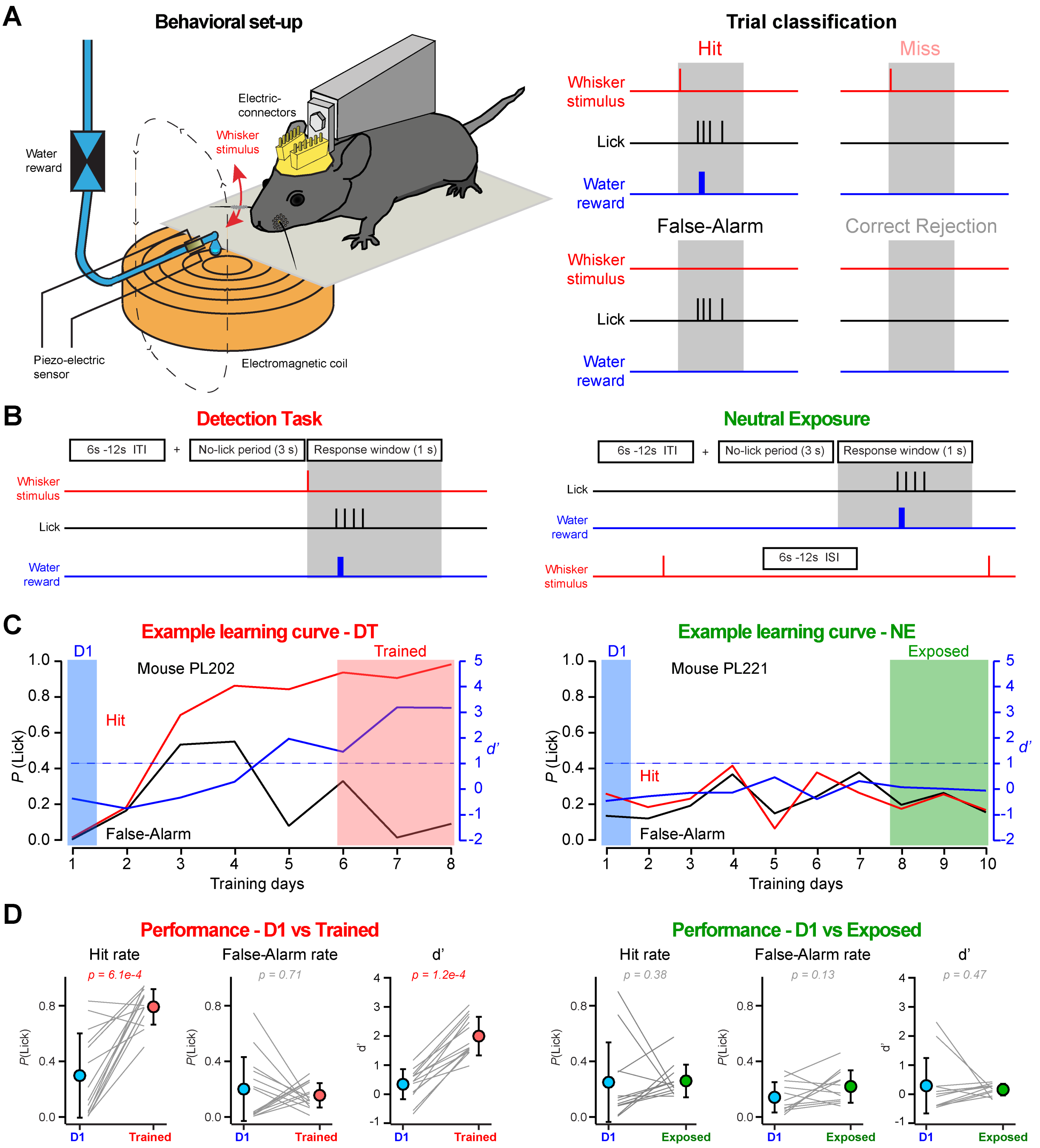

Figure S2. Detection task and neutral exposure

(A) *Left*, Schematic drawing of the behavioral set-up. *Right*, Trial classification. For Neutral Exposure, Licking within the 1 s window following whisker stimulus was scored as Hit, regardless of reward delivery.

(B) Schematic representation of the trial time-line of the two behavioral tasks: detection task (left) and neutral exposure (right). In the detection task, whisker stimulation predicted reward availability. In neutral exposure training, whisker stimulation did not predict reward availability and whisker stimuli were delivered asynchronously at random times.

(C) Example learning curves for a mouse trained in the detection task (*Left*) and a mouse during neutral exposure (*Right*). The red and black curves indicate lick probability (P Lick) on Stimulus trials (Hit rate) and Catch trials (False-Alarm rate), respectively. The blue curve indicates d'.

(D) Performance of all the mice used for LFP recordings in the detection task (*Left*) and neutral exposure (*Right*) for the first session (D1) and mean of the three trained or exposed sessions. Values are mean ± SD. Red *p* values indicate statistically significant differences between D1 and Trained or Exposed (Wilcoxon Signed-Rank test).

Figure S3

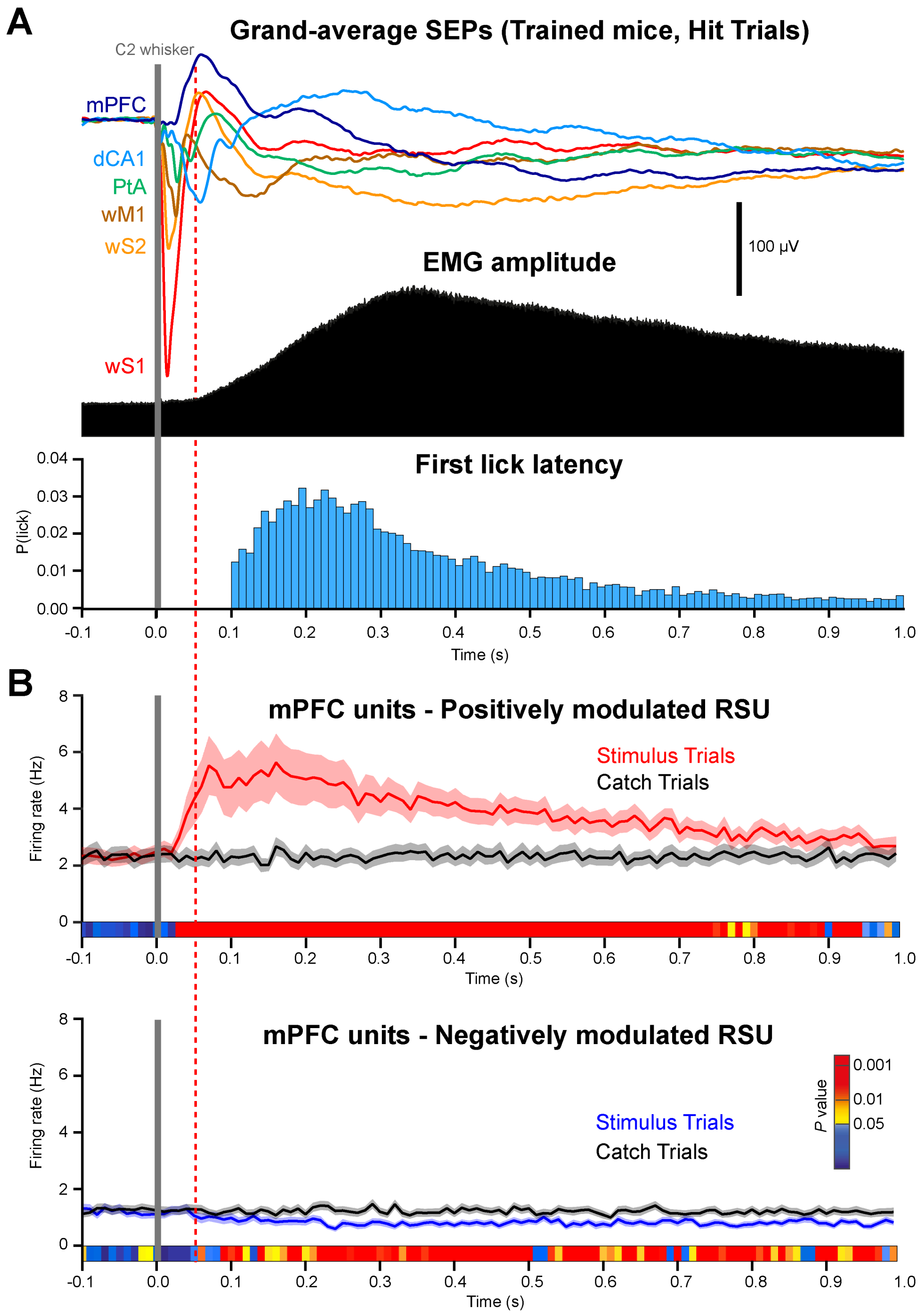

Figure S3. Sensory evoked responses precede EMG activity and licking

(A) *Top*, Grand-average SEPs from the 6 cortical areas computed for Hit trials in trained mice. Premature (<100 ms) lick trials have been excluded. *Middle*, Grand-average EMG amplitude. *Bottom*, Distribution of the first-lick latency. The red dashed line indicates the beginning of the EMG activation.

(B) *Top*, Average PSTHs from mPFC computed for positively (*above*) and negatively (*below*) modulated units for all Stimulus trials (Red and Blue) and all Catch trials (Black). Shaded areas indicate SEM. The bar below the PSTHs indicates color-coded *p* values for statistical test of the difference between Stimulus and Catch trials across time (Wilcoxon signed-rank test Stimulus vs Catch for 10 ms time windows).

Figure S4

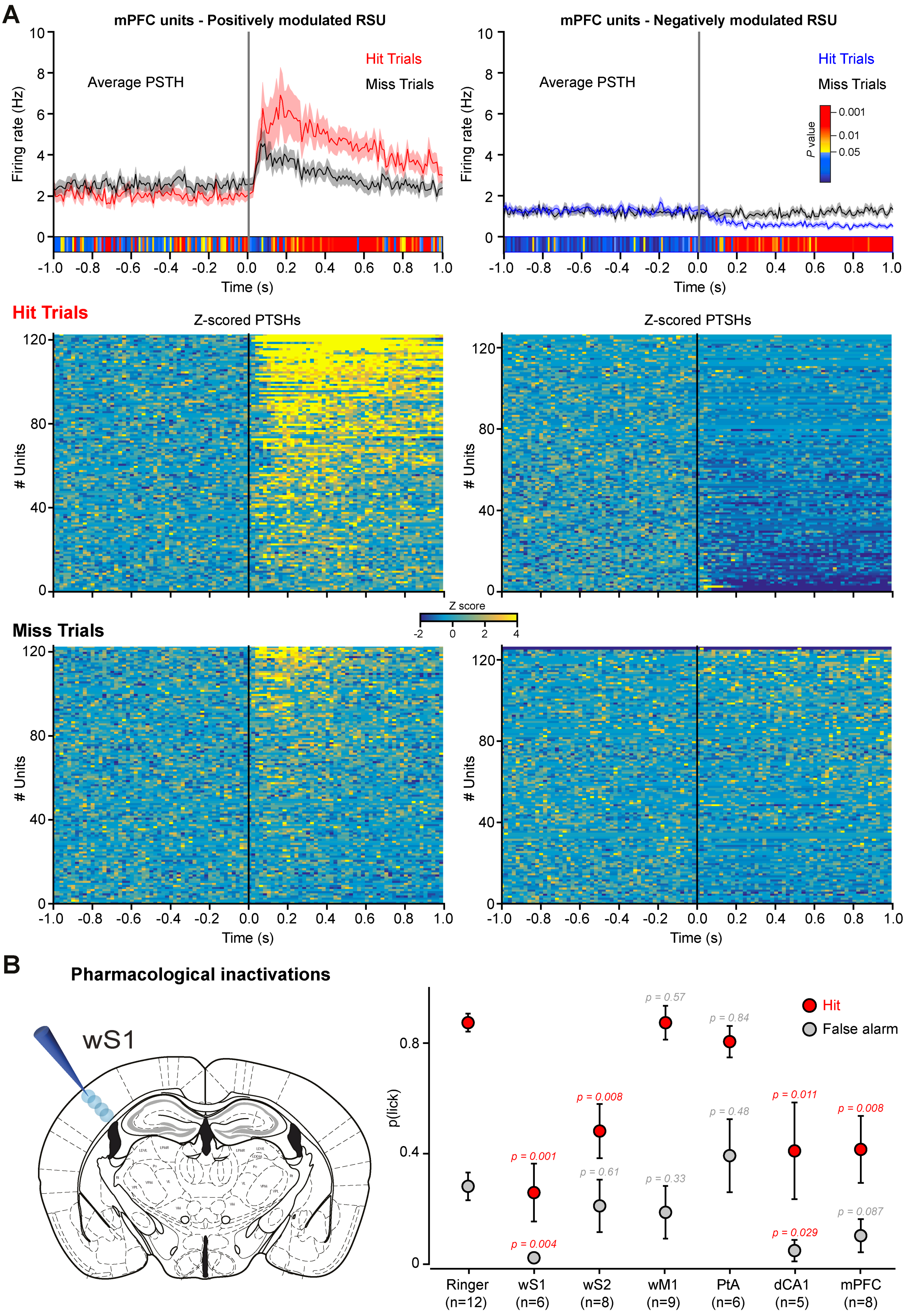
A
mPFC units - Positively modulated RSU
mPFC units - Negatively modulated RSU
Average PSTH
Hit Trials
Miss Trials
Firing rate (Hz)
P value
0.001
0.01
0.05
Time (s)
Hit Trials
Z-scored PTSHs
Units
Z score
Miss Trials
B
Pharmacological inactivations
wS1
p(lick)
Hit
False alarm
p = 0.57
p = 0.84
p = 0.008
p = 0.011
p = 0.008
p = 0.48
p = 0.001
p = 0.61
p = 0.33
p = 0.087
p = 0.004
p = 0.029
Ringer (n=12)
wS1 (n=6)
wS2 (n=8)
wM1 (n=9)
PtA (n=6)
dCA1 (n=5)
mPFC (n=8)

Figure S4. Neuronal activity in mPFC and dCA1 causally contributes to detection task execution

(A) *Top*, Average PSTHs computed for positively (*Left*) and negatively (*Right*) modulated units for Hit (Red and Blue) and Miss (Black) trials. Shaded areas indicate SEM. The bar below the PSTHs indicates *p* values color-coded as indicated in the inset for statistical test of the difference between Hit and Miss trials across time (Wilcoxon Signed-Rank test Hit vs Miss for 10 ms time windows). *Below*, heat-maps of the z-scored PSTHs. The z-scored PSTHs of individual units (20 ms bin size) were aligned to stimulus onset and sorted according to their change in firing rate relative to baseline.

(B) *Left*, Schematic drawing representing the pharmacological inactivation protocol in wS1. *Right*, Effect of pharmacological inactivation (Muscimol, 5 mM, 4 x or 5 x 100 nl) on detection task performance (Hit rate, red; False-Alarm, grey) for each area and comparison with control Ringer injections. The number of mice for each area is indicated in parenthesis. Values are mean ± SD. Statistical significance was assessed by comparing the effect of Muscimol injection with control Ringer injections using Mann-Whitney two sample rank test with a Bonferroni-Holm correction for multiple comparisons: red *p* values indicate statistically significant differences.
